# Bubbles Are Departures from Equilibrium Housing Markets: Evidence from Singapore and Taiwan

**DOI:** 10.1371/journal.pone.0166004

**Published:** 2016-11-03

**Authors:** Darrell Jiajie Tay, Chung-I Chou, Sai-Ping Li, Shang You Tee, Siew Ann Cheong

**Affiliations:** 1 Division of Physics and Applied Physics, School of Physical and Mathematical Sciences, Nanyang Technological University, 21 Nanyang Link, Singapore 637371, Republic of Singapore; 2 Complexity Institute, Nanyang Technological University, Block 2 Innovation Centre, Level 2 Unit 245, 18 Nanyang Drive, Singapore 637723, Republic of Singapore; 3 Department of Optoelectric Physics, Chinese Cultural University, 55 Hwa-Kang Road, Yang-Ming-Shan, Taipei 11114, Taiwan; 4 Institute of Physics, Academia Sinica, 128 Section 2, Academia Road, Nankang, Taipei 11529, Taiwan; East China University of Science and Technology, CHINA

## Abstract

The housing prices in many Asian cities have grown rapidly since mid-2000s, leading to many reports of bubbles. However, such reports remain controversial as there is no widely accepted definition for a housing bubble. Previous studies have focused on indices, or assumed that home prices are lognomally distributed. Recently, Ohnishi *et al.* showed that the tail-end of the distribution of (Japan/Tokyo) becomes fatter during years where bubbles are suspected, but stop short of using this feature as a rigorous definition of a housing bubble. In this study, we look at housing transactions for Singapore (1995 to 2014) and Taiwan (2012 to 2014), and found strong evidence that the equilibrium home price distribution is a decaying exponential crossing over to a power law, after accounting for different housing types. We found positive deviations from the equilibrium distributions in Singapore condominiums and Zhu Zhai Da Lou in the Greater Taipei Area. These positive deviations are dragon kings, which thus provide us with an unambiguous and quantitative definition of housing bubbles. Also, the spatial-temporal dynamics show that bubble in Singapore is driven by price pulses in two investment districts. This finding provides a valuable insight for policymakers on implementation and evaluation of cooling measures.

## Introduction

Housing prices and affordability have always been an intensely debated topic because it involves the livelihood of people. As such, housing market crashes affect much broader cross sections of societies than do stock market crashes. In the most recent case, the subprime housing crisis in the United States (US) was a primer that led to the global financial meltdown in 2008 [1]. Meanwhile, a regional housing bubble of unprecedented scale has been brewing in many Asian cities since 2007. However, since there are no widely accepted definitions for housing bubbles, many Asian governments have denied they are facing such problems [2–4].

Many previous works on detecting bubbles have been done using carefully designed surveys [5, 6], which measures the attitudes of people towards bubble formation using questions, such as those listed in [5]. Another popular and more quantitative method for detecting bubbles include using unit root tests to test for non-stationarity in the fundamental values and asset prices. Using this approach, bubbles have since been discovered in Hong Kong [7], United Kingdom (UK) [8] and several cities in the US [9, 10] for various time periods. More recently, Meng *et al.* computed the correlation matrix of the Housing Price Index (HPI) in 51 US states and found the eigenvalues increasing beyond the maximum predicted by random matrix theory during a period coinciding with the subprime mortgage crisis [11]. Still, others treated housing bubbles as precursors to critical transitions, and have looked for the presence of log-periodic power laws [12–14], as well as spectral reddening, increasing autocorrelation, and increasing variance as we approach the housing market crash [15]. The latter three are generic early warning indicators associated with critical slowing down as a complex system (which housing markets can be classified into) approaches a regime shift [16].

In discussions with policymakers, we often find it difficult to convince them that the signatures we see in the data points correspond to the presence of a housing bubble because there are no unambiguous definitions. We therefore believe that it is important to first understand the housing market equilibrium (if it exists) before we can develop an understanding of housing bubbles. As the price of a real estate can be greatly affected by many external factors, hedonic models are often used in real estate and most notably in the construction of housing indices [17, 18]. While such models have been compared against traditional pricing models (such as the Dutot model) [19], few have tested hedonic models against the empirical home price distribution. A recent work by Ohnishi *et al.* assumed that the price of a house can be treated as a product of a large number of random variable, each corresponding to an external factor. If these random variables have finite means and finite variances, then in the limit of infinitely many factors, the central limit theorem predicts that the home price distribution will converge to a log-normal distribution [20]. However, the empirical distribution of housing prices in Japan has a power-law tail for several years. Similar power-law tails have been observed in the housing price distribution of London [21]. In the hedonic model, this can happen if one or more of the means and variances become infinite.

In general, it is not clear that a power-law tail in the home prices necessarily indicates the presence of a housing bubble. It is important to first establish what kind of distribution we ought to see when the housing market is in equilibrium. As such, our approach is to look at transaction-level housing data over many years, to identify the equilibrium home price distribution. Once this is known, it should be possible to identify out-of-equilibrium features that might correspond to bubbles. Instead of focusing on the differences between cities, we would like to discover universal features of housing market equilibrium and bubbles that are recognizable in all cities.

**Fig 1:**
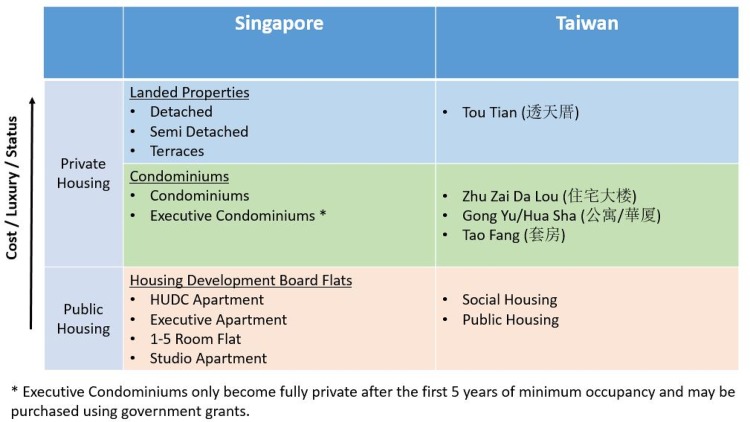
A comparison between housing types in Singapore and Taiwan. Shaded in blue are landed properties, which are detached standalone houses, while those shaded in green are apartments/estates with common amenities that can be shared by residents. Public housing is shaded in red.

Together, Singapore, Taiwan, Hong Kong, and South Korea are known as the Four Asian Tigers. Culturally, Singapore, Taiwan and Hong Kong have a majority of ethnic Chinese and therefore share similar views towards the importance of home ownership [22]. Thus as a proof of concept, we compare the housing markets between Singapore and the Greater Taipei Area (GTA) in Taiwan. In this study, we look across housing type classifications in Singapore and in Taiwan and match the housing types (See Fig 1). We find corresponding housing types for the rich of both the GTA and Singapore. In addition, apartments in estates with amenities, such as condominiums in Singapore and Zhu Zai Da Lous in the GTA appeal to young professionals in both cities. Beyond these similarities, the Singapore and Taiwan housing markets diverge, with subsidized public housing in the former and low cost private housing in the latter making up the rest of the market.

In Fig 1, we show the different housing types in Singapore (SG) and Taiwan (TW). The Singapore housing market is segregated into the landed properties, condominiums and Housing Development Board (HDB) flats. The first two are private properties while the HDB flats are public housing that enjoy subsidies. Taiwan has five housing types—Tou Tian, Zhu Zhai Da Lou, Tao Fang, Hua Sha and Gong Yu. In this report, we compare the landed properties (SG) to the Tou Tians (TW) and the Condominiums (SG) to the Zhu Zhai Da Lou (TW) and Hua Sha/Gong Yu (TW), drawing parallels between the housing types with respect to the type of occupants they appeal to. We also show that the home price distributions in both cities take on a Gibbs-Pareto form, which is well known from the study of income and wealth. Finally, we report the presence of a housing bubble in the private apartment market segment, in both Singapore and the GTA. A preliminary study of the house price distributions of Taiwan can be found in [23].

## Results

### Equilibrium Home Prices

**Fig 2:**
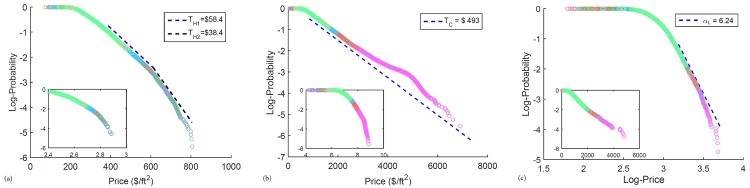
The per-square-foot (PSF) home price CDFs of (a) the HDB flats, (b) condominium apartments and (c) landed properties. The CDFs are plotted in a semilog, semilog and loglog scales respectively. For each of the sub-figure, the alternate distribution is shown in the inset. (a) The HDB flats follow a double exponential distribution with temperatures *T*_*H*1_ = $58.4/ft^2^ and *T*_*H*2_ = $38.4/ft^2^. The second exponential is formed by transactions after 2009. (b) Condominium apartments PSF roughly follows an exponential distribution with temperature *T*_*C*_ = $493/ft^2^, except for a bump between $3000/ft^2^ and $4500/ft^2^ which corresponds to a bubble that started in 2007. (c) The CDF of the landed properties follows a power-law distribution with Pareto exponent *α*_*L*_ = 6.24. For all three sub-figures, the different colors represent a transaction in one of the sectors shown in Fig 3(a).

We start by reporting our findings on the Singapore housing market. Fig 2(a)–2(c) show the cumulative distribution functions (CDF) of price per square foot (PSF) for HDB apartments, condominium apartments, and landed properties. For the condominium and landed properties the data we fitted was from the period 1995 to 2014, whereas the HDB properties data was from 2000 to 2014. We fit part or all of these CDFs to the exponential (Gibbs), power-law (Pareto) distributions, and found that the CDFs of HDB and condominium apartments are best fitted to decaying exponentials (with a log-log plot inset showing a poor fit to the power law). On the other hand, the tail of the landed properties’ CDF is best fitted to a power law (with a semi-log inset, showing poor fit to the exponential distribution). The results of all fits and tests of statistical significance are listed in Section 1 of S1 File.

**Fig 3:**
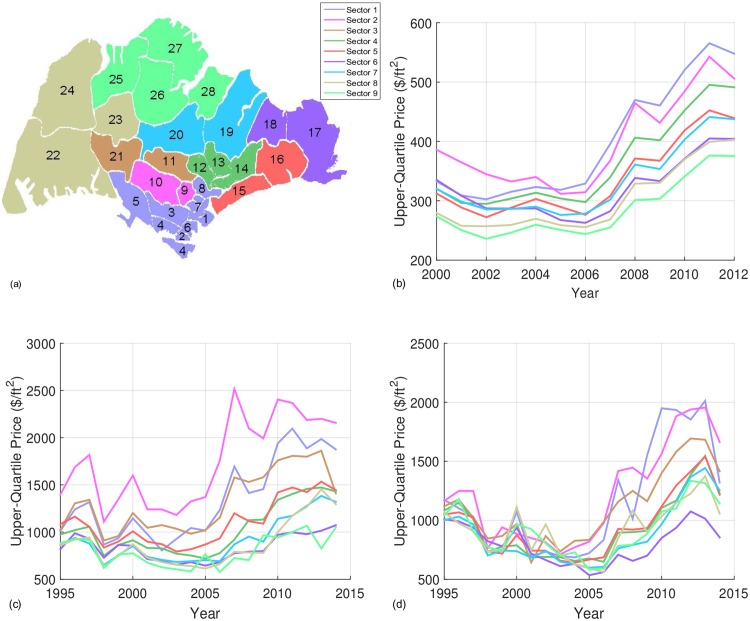
The district/sector map with the upper quartile price of the different property types in Singapore. (a) Map of Singapore with the geographical location of the 28 postal districts (in numbers), segregated into 9 sectors represented by their respective colors. Plots of the yearly upper quartile price for (b) the HDB flats, (c) the condominium apartments and (d) the landed properties, for the 9 sectors in Singapore.

In Section 4 of S1 File we also show the contributions to the CDFs from the various years. For HDB apartments, the yearly contributions to the CDF remain largely the same from 2000 to 2009. This is also the case for condominium apartments and landed properties for the period 1995 to 2006. This suggests that the HDB and condominium segments were in stable equilibria over these periods, and their housing market equilibria are characterized by exponentially decaying CDFs. For the landed properties, the equilibrium distribution is a power law.

### Departures from Equilibrium as Housing Bubbles

In Fig 2b, we see that the condominiums data generally fits well to an exponential distribution with ‘temperature’ *T*_*C*_ = $486/ft^2^, except for a bump that stretches from $3000/ft^2^ to $4500/ft^2^. All units in $ refer to the Singapore Dollars, while all units in NT$ refers to the New Taiwan Dollars. The year-by-year condominium price distributions from 1995 to 2014 are shown in Section 4 of S1 File. From these plots, it is evident that the bump first appeared in 2007, and persisted till 2014. This is in agreement with the upper quartile price spike we see in Fig 3(c). This bump is a large positive deviation from the Gibbs distribution, which suggest a higher-than-expected probability of finding a property selling in that range, than expected from an equilibrium exponential distribution. Didier Sornette calls such statistical features *Dragon Kings* (DK) [24]. To confirm this, we applied the U-test [25] to the condominium CDF using the exponential distribution as the reference. The results of our U-test (see Section 5 of S1 File) suggest that there is at least one DK for prices beyond $3000/ft^2^, which is the beginning of the bump. This observation provides us with a technical and unambiguous definition of a housing bubble, i.e. a dragon-king out-of-equilibrium feature in the home price CDF, and also a statistically rigorous way to test for the presence of the bubble. More importantly, the price distributions of the HDB and landed properties show no positive deviations from their respective equilibrium distributions, which suggest that there are no bubbles in these two market segments.

From the color scheme in Fig 2 we see that the transactions in the bump and beyond are located in the prestigious districts 9 and 10 which make up Sector 2 (magenta) in Singapore (see Fig 3(a)). Situated near the city center, we suspect these properties are ‘investment class’ and susceptible to speculation. To confirm this, we plot the yearly upper quartile price of each sector (represented by their respective colors in Fig 3(a)) in Fig 3(b). We have chosen the 75th-percentile price instead of the mean as we are interested in the tail end of the distribution but at the same time do not want our analysis to be affected by outliers. Prior to the bubble, the movement of prices in all sectors are generally in tandem, as in the case of the HDB and landed properties segments. Following the start of the bubble in 2007, a sudden spike (in 2007) and dip (in 2008) in the condominium upper quartile prices can be seen in several sectors. In particular, the dip which we suspect was in response to the 2008 Global Financial Crisis is deepest for the prime areas (Sectors 1 and 2). Given that this dip is weaker in sectors further from the prime districts, and in some sectors the prices actually rose, this suggests that transactions near or within the prime areas were of a speculative nature, rather than for owner occupation. A repeated sales regression model done by Jiang *et al.* also detected an explosion period from early 2007 to early 2008 [26]. We further verify this explosive growth in the next subsection.

### Spatial-Temporal Dynamics of the Singapore Housing Market

To visualize the spatial and temporal dynamics simultaneously, we constructed a monthly sequence of heat maps to observe how the increase in prices gradually spread across Singapore. All videos of the heat maps are found in S1–S3 Videos, where we see that the home prices for the condominium apartments first started to rise sharply in the prime districts 9 and 10 (Sector 2, magenta), before spreading into the neighboring sectors. This spiking phenomenon is especially clear at the height of the housing bubble in September/October 2007. Then as the bubble slowly relaxed in the prime districts in 2008 and 2009, home price increases continued to spread outwards into districts further away. By the end of 2009, the upper quartile in each sector has risen compared to earlier years for the whole of Singapore. This strongly supports the suspicion that the bubble first formed in districts 9 and 10, possibly from speculative behavior, around 2007. Such spreading effects arising from Sector 2 are also observed in the heat maps of the Landed and HDB segments (see Section 4 of S1 File), but the pulsatile dynamics are not observed in both the HDB flats and the landed properties, where there are no bubbles.

The spatial and temporal dynamics for all three housing types show that price increase generally starts from the prime districts before spreading out to the peripheral districts. Across housing types, we see that the price increase was led by the condominiums which peaked in 2007, followed by the peaks of the HDB (2011) and landed properties (2010-2012). We also found a second exponential part in the HDB price distribution appearing after 2009, after the bubble formed in the condominiums. Therefore, we suggest that these purchases are likely to have been made by those who are affected by the condominium housing bubble. However, more data will be needed to better understand this phenomenon.

### Comparison between Singapore and Taiwan Housing Markets

**Fig 4:**
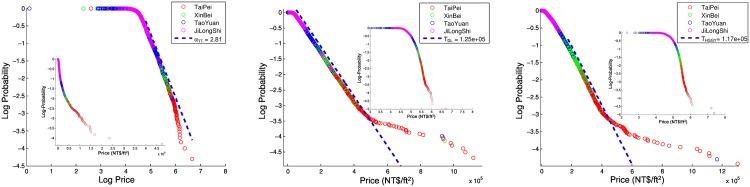
The PSF CDFs of the different property types in the GTA. The CDFs of the (a) Tou Tian (b) Da Lou and (c) Gong Yu and Hua Sha, shown in loglog, semilog and semilog scales respectively. For each plot, the alternative distribution is shown as the inset. We see that (a) the Tou Tian is power-law distributed with a Pareto exponent of *α*_*TT*_ = 2.81, (b) the Da Lou in the GTA largely follows an exponential distribution with *T*_*DL*_ = NT$1.25 × 10^5^ and (c) the Gong Yu/Hua Sha in the GTA follow an exponential distribution with *T*_*HSGY*_ = NT$1.17 × 10^5^. For (b) the Da Lou and (c) Gong Yu/Hua Sha, we observe large positive deviations in the tail of the distribution that indicates the presence of a bubble.

Fig 4 shows the fits to the PSF CDFs of the different housing types in the GTA of Taiwan. We see that the Tou Tian is Pareto distributed (*α*_*TT*_ = 2.81), and we can see this as a straight line in the loglog plot. Both the Zhu Zai Da Lou and Gong Yu/Hua Sha follow exponential distributions for most part of their bodies, but deviates positively at their tails.

The Tou Tian are detached houses similar to landed properties in Singapore. Ownership of such houses is symbolic of one’s status and hence these houses are usually owned by the rich in the respective cities. The Zhu Zhai Da Lou and Tao Fangs are very similar to the condominiums and shoe box condominiums in Singapore. These homes usually have common amenities such as swimming pools and other recreational facilities, appealing to the upper-middle class. The Gong Yu and Hua Sha which resembles more like the HDB flats in Singapore follows an exponential distribution.

As we can see, the equilibrium distributions of corresponding housing types are the same between the Singapore housing market and Taiwan housing market. We observe that the more expansive and desired housing type is Pareto distributed in both Singapore and in the GTA. The upper-middle class housing types are exponentially distributed, with a possible dragon king in the Singapore condominiums and in both the GTA’s Zhu Zhai Da Lou and Gong Yu/Hua Sha. This comparative study between Singapore and Taiwan reminds us of a recent study conducted on the Housing Price Index (HPI) of the top ten key cities of China [27]. It is discovered the top tier cities, Beijing, Guangzhou and Shenzhen, collectively referred to as a club, are key drivers in the rise of the HPI index in China. Similarly, we can expect the cities in the GTA to be the driver in Taiwan home prices. However, here in this study, we used raw transaction prices as compared to aggregated HPIs of different cities in the GTA.

## Discussion

### Income, Wealth, and Home Price

Singapore tightly regulates the ownership of properties by foreigners. Landed properties can only be bought by Singaporeans and Permanent Residents (PRs) as these are “considered a special class of property that Singaporeans aspire to own” ([28], para. 30). On the other hand, there are no restrictions on foreign ownership of condominiums. However, in recent years rules have been put in place to strongly discourage speculative buying. Finally, the heavily-subsidized HDB flats can only be owned by Singaporeans and eligible PRs. Regulations governing ownership of specific housing types in Singapore can be found in the Section 2 of S1 File.

Therefore, there should be no surprise that in Singapore, the rich and higher-income group tend to live in landed properties, while the middle class lives in condominium apartments and HDB flats. Though the housing market is officially classified into three separate segments (see Fig 3), this Gibbs-Pareto distribution of home prices mirrors the Gibbs-Pareto distribution of income/wealth distribution. This exponential body-power-law tail structure of wealth/income distribution has also been verified by data for US and UK [29, 30] and Japan [31]. This distribution follows naturally from a model of wealth produced by additive and multiplicative processes [32, 33]. At present, there is no mechanistic theory of home prices, nor is there theoretical arguments suggesting a connection between home price and wealth. Since housing is a significant component of a household’s wealth, such a close relationship is naturally expected, but not yet verified in real-world data. Going through London’s home price data, MacKay was one of the first to suspect a connection between the two distributions [21]. We also suspect such a relationship in Singapore. However, this is tricky to test as the income and wealth data in Singapore is not publicly available. This is because they are collected under the Statistics Act and thus confidentiality is protected under the act. Therefore, we use as proxy the Forbes top 50 wealthiest Singaporeans list [34] to estimate the Pareto exponent of wealth to be *α* ≈ 2.2 (see Section 2 of S1 File). For the case of Taiwan, we found that the income distribution for the whole of Taiwan follows a Gibbs-Pareto distribution with a Pareto exponent of *α* = 5.0 (see Section 2 of S1 File), while the wealth distribution for Taiwan, as estimated from the Forbes top 50 wealthiest Taiwanese list [35] has Pareto exponent *α* ≈ 2.5. We see therefore that the Pareto exponent *α*_*TT*_ = 2.82 of the Tou Tian PSF CDF in the GTA is comparable to the Pareto wealth exponent of *α* ≈ 2.5. The small discrepancy can be explained if we take into account the fact that much of the wealth in Taiwan is concentrated in the GTA, and hence we expect the wealth distribution within the GTA to have a fatter tail. On the other hand, the Pareto exponent *α*_*L*_ = 6.24 for landed properties in Singapore is significantly different from the Pareto wealth exponent of *α* ≈ 2.2. We suspect this is due to a combination of being a small market and being tightly regulated, and have started a followup study linking wealth and home prices.

### Bubble and Bubble-Like Features in Home Price CDF

We find three ways the home price distribution can change with time. First, even after discounting the housing price with the CPI, we see the tail of the price distribution is extending towards higher price levels. However, this tail extension is either along the equilibrium distribution, or falling slightly below the equilibrium distribution. This can be seen as a general upward trend in the upper quartile price for all the HDB, condominiums and landed properties in Singapore (Fig 3). We do not know whether this also happens for the GTA, since we only have two years worth of data. However, since there is no systematic positive deviations from the equilibrium distribution, we do not consider such cases as bubbles.

Second, we observe positive deviations to the equilibrium distribution, as seen in the condominiums (SG), the Zhu Zhai Da Lous (TW) and Gong Yu/Hua Sha (TW). These deviations are possible DKs which might correspond to a bubble. While this can also be seen as a sharp rise in the upper quartile price, in such plots it is difficult to distinguish DKs from a simple tail extension. In such cases, a suitable statistical test such as the U-test [25] must be carried out.

Finally we may also find an upward flexing of the power-law tail in the housing price distribution. Most power laws in physical systems are known to be robust. For example, the exponent in the Gutenberg-Richter’s (GR) Law for earthquakes is always very close to 1, no matter which seismically active region we are talking about. However, Ohnishi et al. [20] observed changes in the power law exponents during the years when Japan was experiencing a property bubble. From our preliminary study of the London housing market, we also observe the same upward flexing of the flats/apartments distribution in London from the period 2012 to 2014. We consider such cases as bubbles, as these case may be a more extreme case of the second phenomenon (positive deviations to empirically fitted distribution) elaborated in the previous paragraph. In fact, upward flexing of the GR Law have been found to precede large earthquakes [36].

### Cause of Singapore Housing Bubble

Just like the US housing bubble was the result of credit easing encouraged by the government, regulatory easing in Singapore is also the likely cause of the bubble formation in the condominiums but not in the other segments. HDB properties and landed properties remain highly regulated, making speculative investments difficult. Had such restrictions been waived as well, these two segments can potentially participate in the housing bubble as well, as demonstrated by landed properties on Sentosa, a small island south of the main island of Singapore, where large price swings have been observed. In a sense, regulatory oversights successfully prevented a housing bubble in the HDB and landed housing market segments.

## Data and Methods

All the raw data files in this section can be obtained in whole or in part through instructions and links provided in S2 File

The Singapore housing price data is obtained from two sources. The Urban Development Authority (REALIS—Real Estate Information Systems) database captures transactions of private properties dating back to January 1995 [37]. The information provided for each transaction includes the sale price, type of housing, the price per square foot ($/ft^2^) as well as the location of the property. The parcel type (land/strata) and lease type (freehold/99-years/999-years) is also reflected in the data. The HDB data is obtained from the official national statistics website of Singapore [38]. It consists of the town in which the property is located, the flat type (number or rooms), date of transaction, the size of the property as well as the transaction price of the property.

The data for the property transaction price of the GTA can be obtained from the Department of Land Administration, Ministry of the Interior, Republic of China [39], and consists of the transaction data of the price, floor area, type of housing, city and district of the property for the entire Taiwan from 2010 to 2012.

Spatially, Singapore can be segregated into 9 different sectors consisting of a total of 28 districts. The map illustrating the different districts and sectors is shown in Fig 3(a). The GTA of Taiwan consist of Taipei City, New Taipei City, Keelung City and Taoyuan City, which forms the Greater Taipei-Keelung-Taoyuan metropolitan area.

For a meaningful comparison of the data across different years, all home prices are discounted using the consumer price index (CPI) of Singapore/Taiwan. The historical CPI of Singapore is obtained from the Singapore Statistics website [40], while the historical CPI of Taiwan is obtained from National Statistics, Republic of China [41]. The year’s simple average CPI is used to discount the price to the base year, 2014 (Singapore) and 2011 (Taiwan). We have chosen the CPI over other indicators as a basis for discounting because it reflects the overall spending power of an individual/household relative to the base year.

The CDFs of respective data sets are then fitted using both an exponential and a Pareto distribution. In the exponential fit, the per square foot (PSF) price, *x*, is fitted using the distribution,

$$\begin{array}{c} P\left(X=x\right)\propto \exp\left(-\frac{x}{T}\right), \end{array}$$ (1)

where *T* is the temperature of the Boltzmann-Gibbs distribution. In the Pareto fit, we use,

$$\begin{array}{c} P\left(X=x\right)\propto x^{-\alpha } \end{array}$$ (2)

where *α* is the the Pareto exponent. A Maximum Likelihood Estimation (MLE) method is used to estimate the ‘temperature’, *T* of the exponential distribution, and also the exponent *α* of the Pareto distribution, and the threshold *x*_*min*_ above which this distribution holds.

We then run *p*-tests to check the statistical significance of the fits to a (a) Pareto distribution, and a (b) Exponential distribution. The *p*-test is conducted using the Clauset-Newman algorithm for both distributions [42]. A *p*-test value of *p* > 0.05, which is half the value suggested by Clauset and Newman is used as an acceptance criterion. We also visually inspect the plots to identify the presence of any significant outliers, which we defined as positive deviations to empirically fitted data and upwards flexing of the tail (refer to Section 4 of S1 File).

Next, the U-test is used to confirm if the suspected outlier(s) are DKs. We highlight the working mechanism of the U-test here, but a more detailed explanation can be found in original paper by Sornette and Pisarenko [25]. Suppose we know the CDF of a set of data *X* = {*x*_1_, *x*_2_, …, *x*_*n*_}, where this set is ordered in descending order. If we input the values in the set *X* back into the known CDF, we will obtain a set *U* which is distributed by the standard uniform distribution, where 0 < *u* < 1. The *k*-th ranked sample of the set *U* is distributed with probability,

$$\begin{array}{c} f_{k}\left(u\right)=\frac{n!}{(n-1)!(k-1)!}u^{k-1}{\left(1-u\right)}^{n-k}. \end{array}$$ (3)

The probability that the observed *x*_*k*_ is an overestimate of the true distribution is (from the empirical observation), or *p*-value, can be given by a sum of all possible values from the empirically fitted values to the maximum value, which equals to unity. In the continuous limit, we have,

$$\begin{array}{c} p\left(x_{k}\right)=\frac{n!}{(n-k)!(k-1)!}\int_{Z_{k}}^{1}u^{k-1}{\left(1-u\right)}^{n-k}du=1-betainc\left(Z_{k},n-k+1,k\right), \end{array}$$ (4)

where *Z*_*k*_ is the empirical observed cumulative probability, *betainc* is the normalized incomplete Beta function and *F*(*x*_*k*_) obtained using the empirically fitted parameters. This measures the probability of the empirically observed sample deviating from its fitted distribution.

Finally, we locate the housing transactions corresponding to the outliers. Using this information, we analyze the similarity between anomalous transactions spatially and temporally. We present this information in the form of a heat map of housing prices in Singapore. For each sector, we obtained the 75th-percentile price of all the housing transactions in a month. The 75th-percentile prices for each region and time frame is color coded, with red signifying prices closer to the maximum observed value throughout the years and blue signifying prices closer to the minimum observed value throughout the years. By observing the price change in both space and in time, we are able to clearly see the emergence and evolution of the bubble.

## Supporting Information

S1 File The Supporting information for Bubbles are Departures from Equilibrium Housing Market. This file contains additional information on the housing policies in Singapore and Taiwan as well as significance testing of the results in the main paper.
(PDF)

S2 File Data Sources and Specific Instructions to obtain the Data used. This file contains additional information on the data sources used. The specific URLs and methods to obtain the data in parts or in whole is also contained within this document.
(PDF)

S1 Video The heat map of the HDB flat transactions from Jan 2000 to Feb 2012. (AVI)

S2 Video The heat map of the condominium transactions from Jan 1995 to Dec 2014. (AVI)

S3 Video The heat map of the landed properties transactions from Jan 2000 to Dec 2014. (AVI)
