# Supplementary material for: Bubbles Are Departures from Equilibrium Housing Markets: Evidence from Singapore and Taiwan
Source: PLoS One. 2016 Nov 3;11(11):e0166004. doi: 10.1371/journal.pone.0166004 (PMC5094767; doi:10.1371/journal.pone.0166004)
Supplement: S1 File — This file contains additional information on the housing policies in Singapore and Taiwan as well as significance testing of the results in the main paper. (PDF) [file pone.0166004.s001.pdf]

Bubbles are Departures from Equilibrium Housing Markets: Evidence from Singapore and Taiwan
Supplementary Information

1 Housing in Singapore and Taiwan

There are 3 main housing types in Singapore, namely, (i) the Housing Development Board (HDB) flats, (ii) the condominium apartments and (iii) landed properties. HDB flats are typically high-rise public housing which majority of the average Singaporean live in. Condominium apartments cater more to the upper middle class and have common facilities such as swimming pools, gymnasiums and entertainment rooms that the residents have access to. The landed properties are properties that are detached or semi-detached terraces and bungalows. These properties also usually have a small front/back yard and porch attached.

Generally, the Singapore government provides subsidies to first-time Singaporean home owners who purchase HDB flats and executive condominiums (ECs) apartments. The HDBs is a public housing scheme which is strictly regulated by the Singapore government. Only Singaporeans and Singapore Permanent Residents (SPRs) are allowed to purchase HDB flats. With effect from August 2013, SPR must wait for 3 years after obtaining SPR status before purchasing HDB flats. For most new HDB flat purchases, there is a minimum occupancy period for which the owners cannot own other HDB flat or private property in Singapore or overseas [1].

Condominiums and landed properties are collectively known as private properties in Singapore. This is with the exception of new ECs that have not fulfilled the minimum occupancy period. The condominiums (including ECs that have passed the minimum occupation period) can be freely bought and sold with very minimal restrictions. The landed properties in mainland Singapore however, cannot be owned by foreigners. Foreigners can however, own landed properties on Sentosa island only if it is owner occupied [2].

In Taiwan, most housing properties are privately owned, with only a few exceptions of public housings owned by the government. When one buys a house, an apartment or a studio, he also owns part of the land permanently where the building is located. This is different from places like Hong Kong and China where one only owns the land for a certain period of time.

There are several housing types in Taiwan. Tou Tians are old Taiwanese style detached houses. These houses do not have front or back yards like the detached houses in the US and they have usually existed for at least several decades. There are a few newer types of detached houses or town houses that are built in recent years, e.g. in the suburbs of (Greater Taipei Area) GTA. The prices of Tou Tians vary a lot, depending on their locations. Tou Tians in the GTA are usually expensive even if they are very old, simply because of the land owned by the owner.

Although Zhu Zai Da Lou, Gong Yu and Hua Sha all have apartments and/or studios (Tao Fang), there are differences among them. Zhu Zai Da Lou are high rise buildings (usually much more expensive and newer) and many of them are located in communities that have good public facilities. In some cases, e.g., in Taipei, they belong to the tail of the power law in the distribution. Hua Sha are buildings with elevators (usually under 10 floors and newer). Gong Yus are usually buildings with 5 or fewer floors. They are older (20 years or more) and without any public facilities such as elevators. Hua Sha and Gong Yu are usually cheaper. Most middle class people can only afford to buy an apartment in Hua Sha or Gong Yu in recent years.

2 Maximum likelihood estimate (MLE) and p-Test

Table 1: The PDF of the exponential and power law distribution with their respective normalizing constants. The MLE parameters for each distribution is shown in the last column of the table. The angled brackets denotes the average value.

Distribution	PDF	Estimated Parameter
Exponential	$\frac{1}{T}\exp\left(\frac{x_{min}-x}{T}\right)$	$\hat{T} = \bar{x} - x_{min}$
Power Law	$\frac{\alpha-1}{x_{min}}\left(\frac{x}{x_{min}}\right)^{-\alpha}$	$\hat{\alpha} = 1 + \left\langle \ln \frac{x}{x_{min}} \right\rangle^{-1}$

All power laws and exponential distributions in the main document is fitted using the distribution functions shown in **Table 1**. We show the derivation of the maximum likelihood estimate (MLE) parameter for the exponential distribution below. The derivation for the power law distribution follows a similar process and can be found in the paper by Clauset *et. al* [3]. The probability distribution function (PDF) of the exponential distribution is,

$$P(X = x) = A \exp\left(-\frac{x}{T}\right), \tag{1}$$

where A is a normalizing constant which can be determined by apply the normalizing condition,

$$\int_{x_{min}}^{\infty} P(X = x)dx = 1. \tag{2}$$

We solve for normalizing constant,

$$A = \frac{1}{T}\exp\left(\frac{x_{min}}{T}\right) \tag{3}$$

The likelihood function is,

$$L\left[P\left(X = x|\hat{T}\right)\right] = \Pi_{i=1}^{N} P\left(X = x_i|\hat{T}\right), \tag{4}$$

where L is the likelihood function given the parameter $\hat{T}$. A necessary condition for the likelihood function to be maximum is,

$$\frac{\partial L}{\partial \hat{T}} = 0. \tag{5}$$

Since the natural logarithm is a monotonically increasing function, an equivalent necessarily condition for L to be a maximum is

$$\frac{\partial \log(L)}{\partial \hat{T}} = 0, \tag{6}$$

which can be solve to get,

$$\hat{T} = \bar{x} - x_{min} \tag{7}$$

where $\bar{x}$ is the mean value $\bar{x} = \Sigma x_i/N$. We can also easily check that the second derivative test gives $\frac{\partial^2 \log(L)}{\partial \hat{T}^2} = -\frac{N}{\hat{T}^2} < 0$ which confirms the condition in (7) yields the maximum likelihood value. All p-test are done using MATLAB © and we report all p-values in **Table 2** below. However, we omit the p-values of those data set which might contain significant outliers known as Dragon Kings (DKs) here and explain them in greater details in **Section 5**.

Table 2: The p-test values for each of the fitted distribution. Due to the presences of Dragon Kings (DKs) in the the distribution of the condominiums, Da Lou and Gong Yu/Hua Sha, the p-test is not conclusive for these distributions.

Data	Distribution	p-value	Remarks
Singapore HDB	Double Exponential	0.10	
Singapore Condominiums	Exponential	-	DKs
Singapore Landed	Pareto	0.62	
Taiwan Toutian	Pareto	0.02	
Taiwan Da Lou	Pareto	-	DKs
Taiwan Hua Sha/Gong Yu	Pareto	-	DKs
Singapore Wealth (Forbes)	Pareto	0.200	Finite Size
Taiwan Wealth (Forbes)	Pareto	0.025	Finite Size
Taiwan Income	Gibbs-Pareto	0.513	

3 Income and Wealth

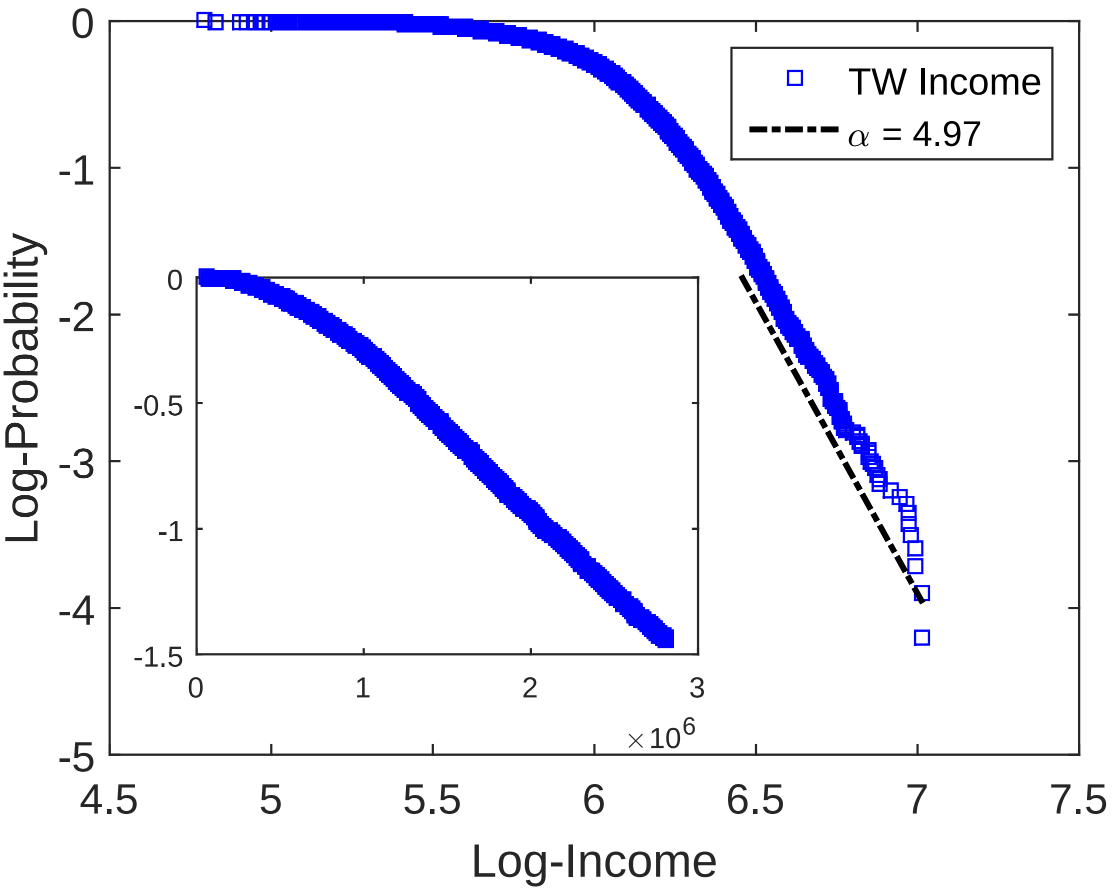

Figure 1: The CDF of the Taiwan wealth distribution on a log-log plot following a straight line with $\alpha = 4.97$. In the inset, we show the semi-log plot of the same data plotted up to x_{min}. We see that the body of the distribution generally follows an exponential distribution which is seen as a straight line on the semi-log plot.

We fit the cumulative distribution function (CDF) of the Forbes top 50 richest Singaporeans and Taiwanese [4, 5] respectively to a power-law and use it to estimate the exponent of the wealth distribution. The income distribution of Taiwan is obtained from [6, 7] and plotted in **Figure 1**. In the main figure, we see that the distribution is a straight line so follows a power-law distribution, while in the semilog inset, it is a straight line up to x_{min} which also show that it has an exponential body. This is in agreement with the Gibbs-Pareto distribution observed in the United Kingdom and United States [8, 9]. However, the exponent for the power-law regime of the Taiwanese income distribution is much higher than those observed in the other parts of the world.

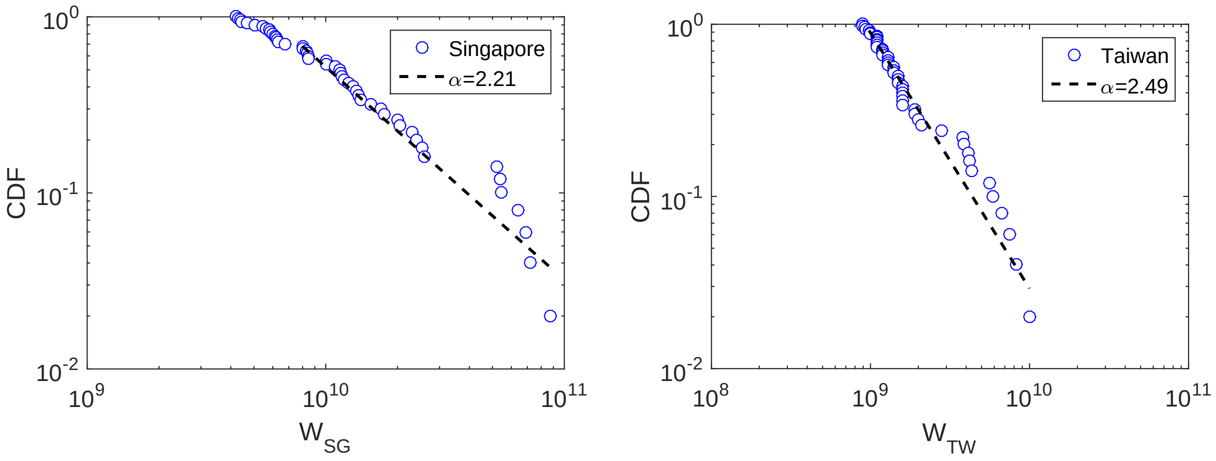

Figure 2: The wealth distribution of the top 50 richest person in (left) Singapore and (right) Taiwan. The p-value of the fit is given in **Table 2**

4 Year-by-Year plots and Heat Maps

The year-by-year plots for each housing types in Singapore are obtained by plotting the yearly contributions to the CDF. The yearly contribution to the HDB CDF generally follows a straight line which suggests that it is in an equilibrium from the period 1995 to 2009. The second exponential regime only appears after 2009. For the condominium CDF contribution, we can see that the distribution is a stable exponential before 2007. A bump above the empirically fitted distribution only appears after the year 2007 and persists beyond that indicates a bubble forming in the condominium segment of the Singapore housing market. The landed properties is a straight line in the log-log plots for all the years of the data set which shows that the landed properties in Singapore is power law distributed and stable throughout the period 1995 to 2014.

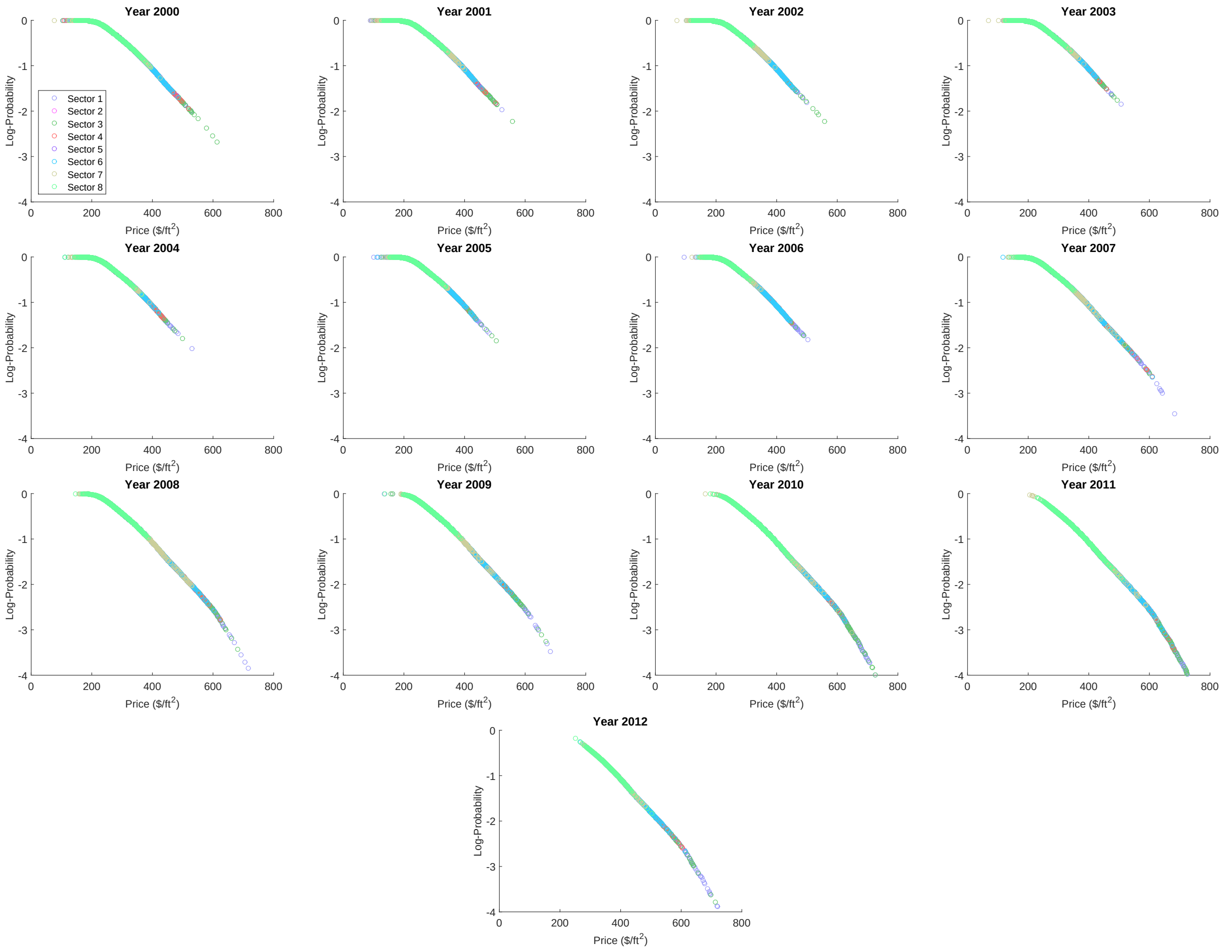

Figure 3: HDB CDF Year By Year Plots from 2000 to 2012

The price of the various housing types can be studied in space and time simultaneously using heat maps. To obtain the heat maps, we store the monthly upper-quartile price for each sector. Using the colormap function (with "jet" color scheme) in MATLAB ©, we set the lowest and highest price observed in the data set for each of the housing types to represent blue and red respectively. Next, each of the upper quartile price stored is transferred into a color between red and blue using linear regression. Each frame in the heat map movie then shows the upper quartile price of the different sectors, for a particular housing type. Here, we show selected frames for each of the different housing types for the purpose of illustrating the spatial-temporal price dynamics in the Singapore housing market. For the full duration of the data set, please refer to the attached videos in the Supporting Information.

From the heat map, we can see that the price increase started with a spike in sector 2. This price increase then spreads to the neighboring districts in the following months. This general dynamics applies to all three different housing types. From the heat map, we are also able to detect the bubble emerging in the condominium segment of the Singapore housing market. For the HDB flats and landed properties, an increase in price is quickly dissipated into the other sectors. On the other hand, we can evidently see that the condominiums in sector 2 was much higher priced than the other sectors during the peak of the bubble in September 2007.

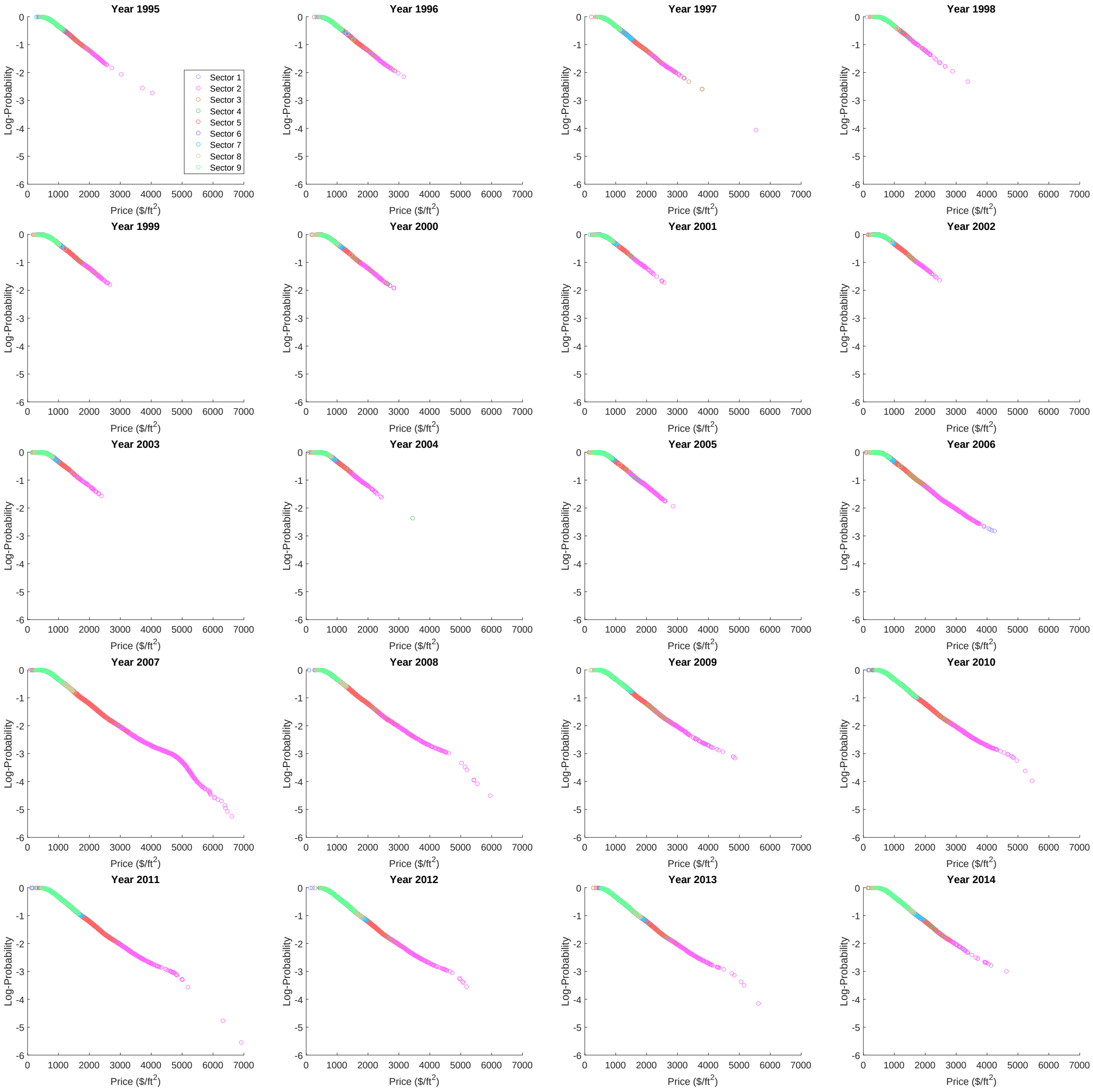

Figure 4: Condominium CDF Year By Year Plots from 1995 to 2014

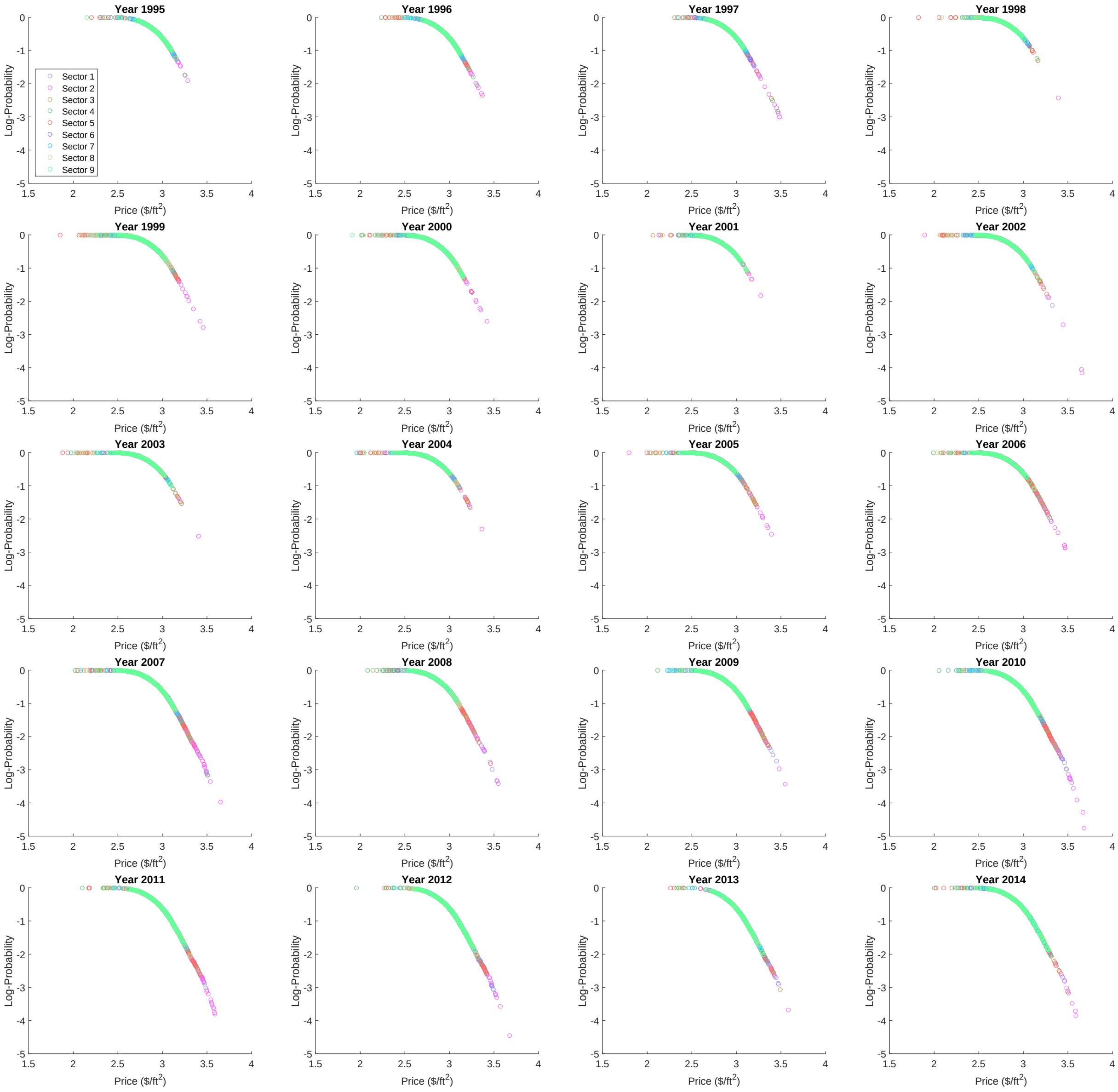

Figure 5: Landed properties CDF Year By Year Plots from 1995 to 2014

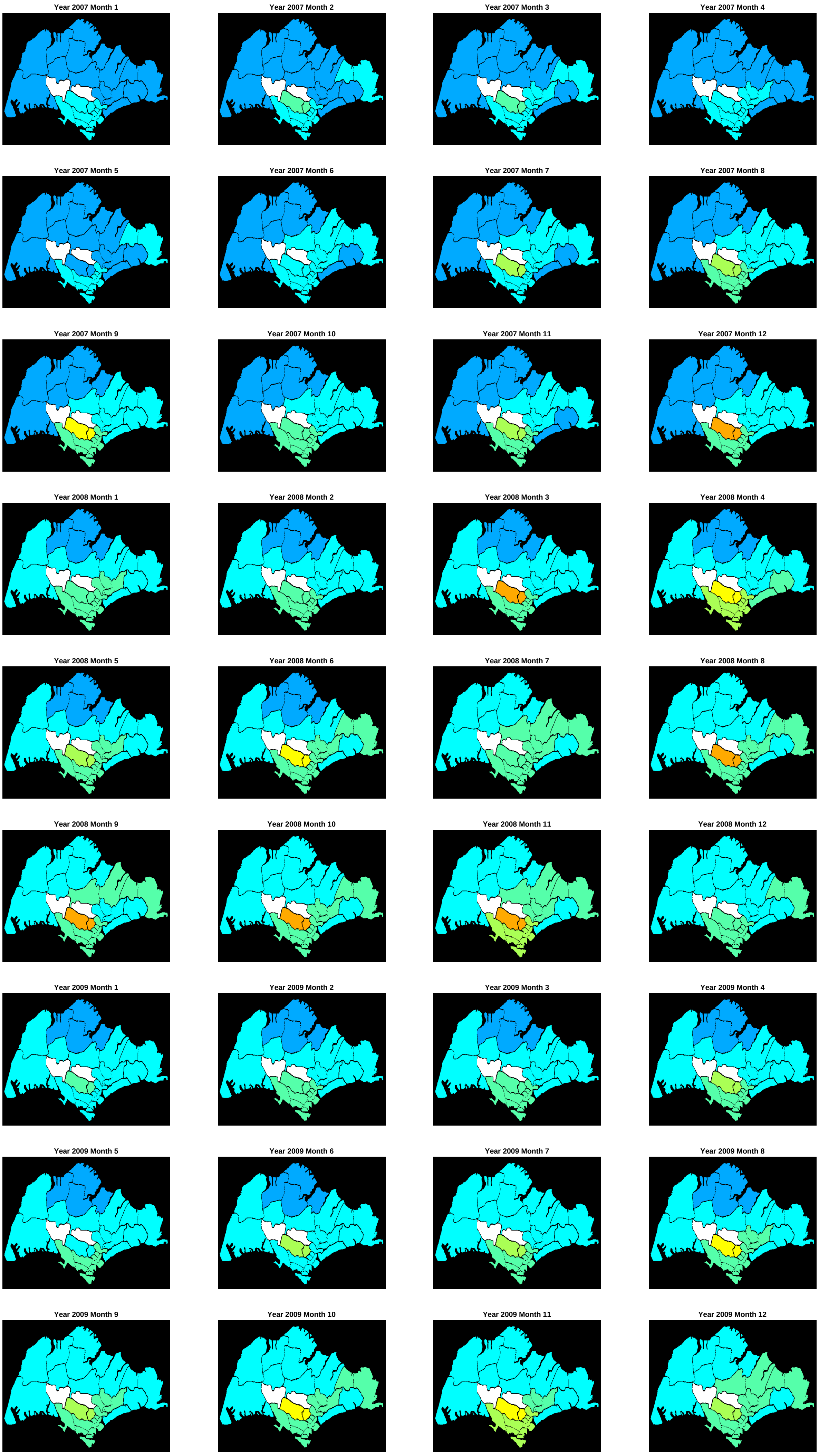

Figure 6: HDB Heat Map from 2007 to 2009

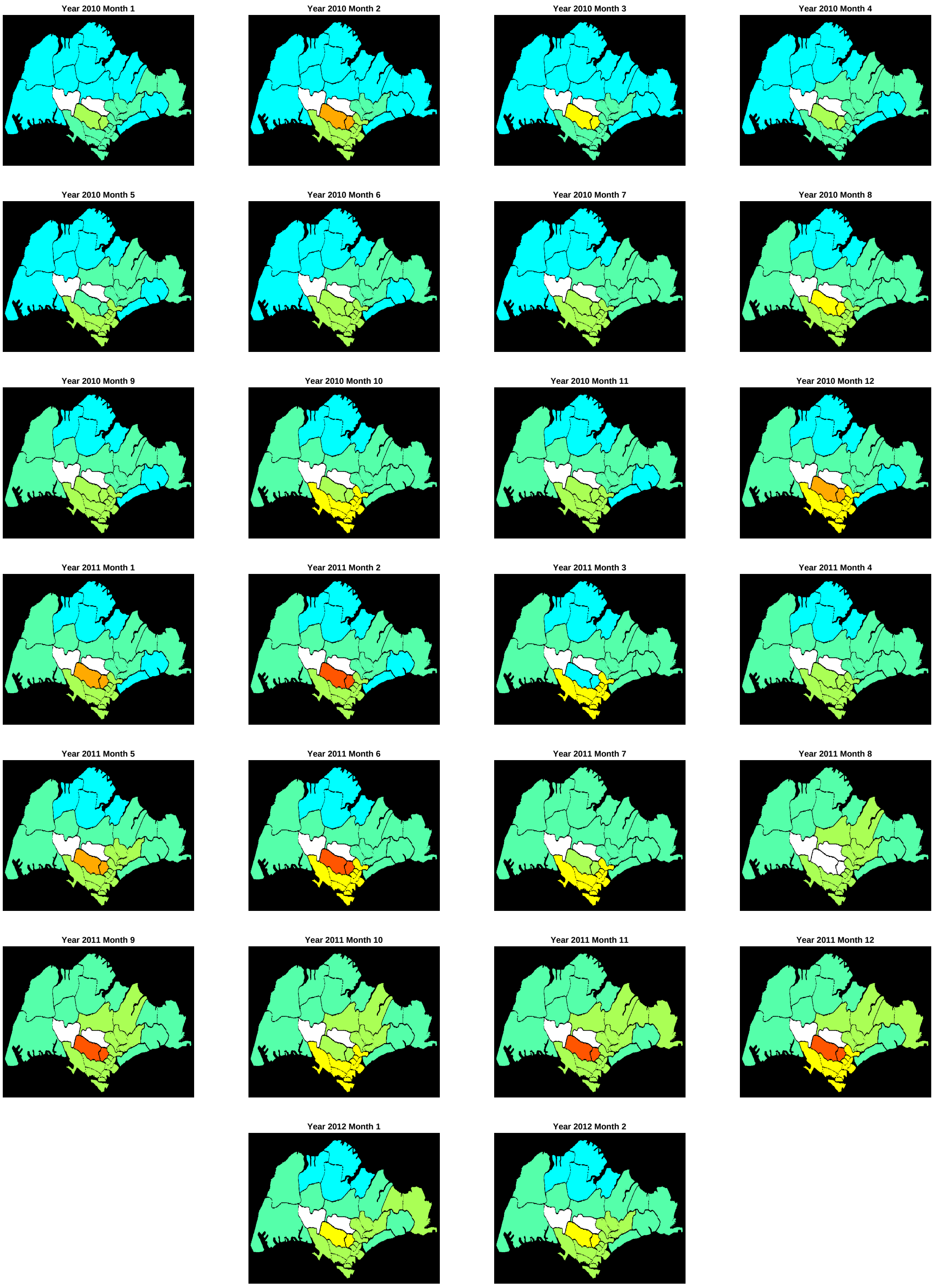

Figure 7: HDB Heat Map from 2010 to Feb 2012

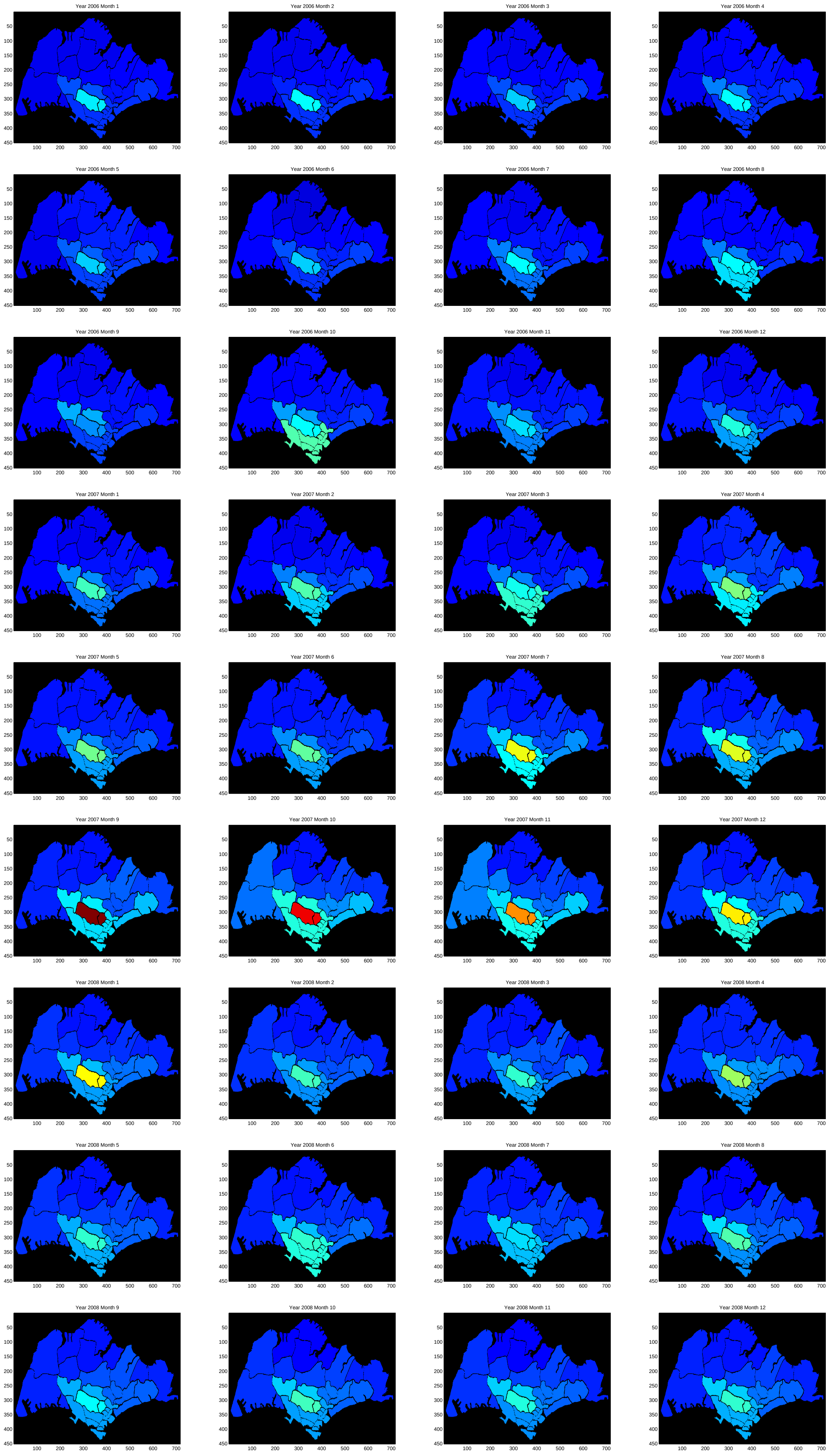

Figure 8: HDB Heat Map from 2006 to 2008

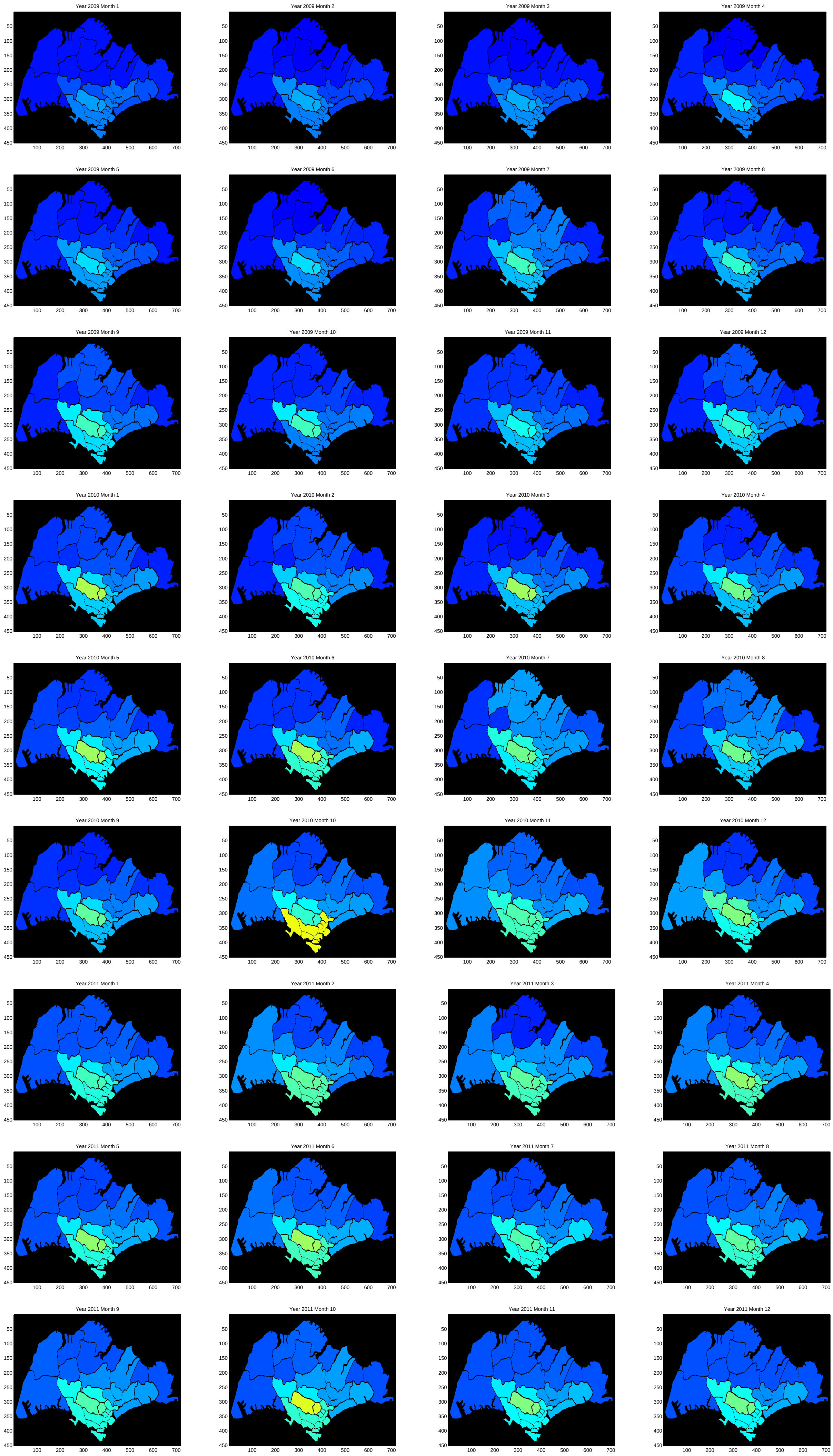

Figure 9: Condominium apartment Heat Map from 2009 to 2011

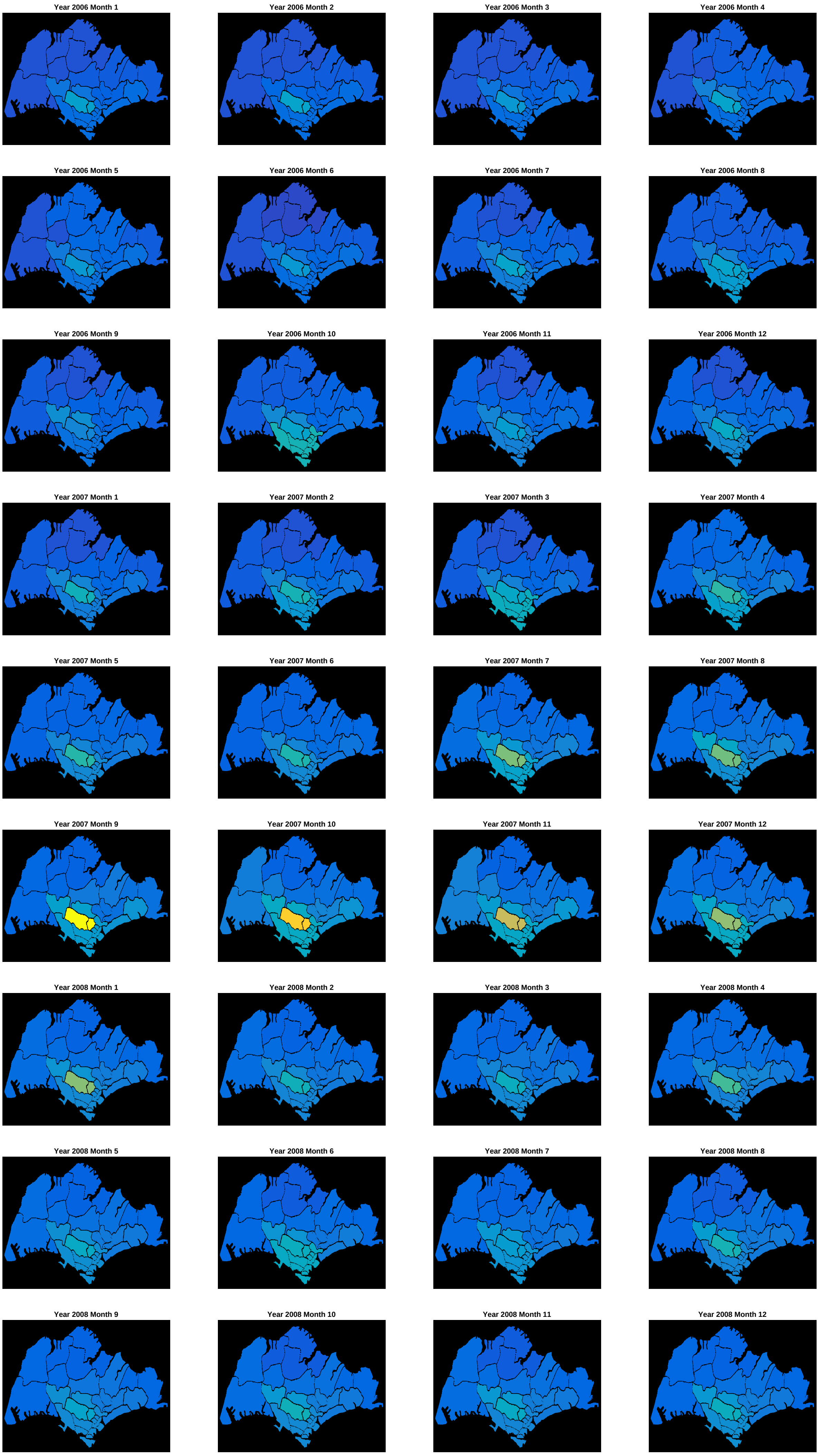

Figure 10: Landed Heat Map from 2006 to 2008

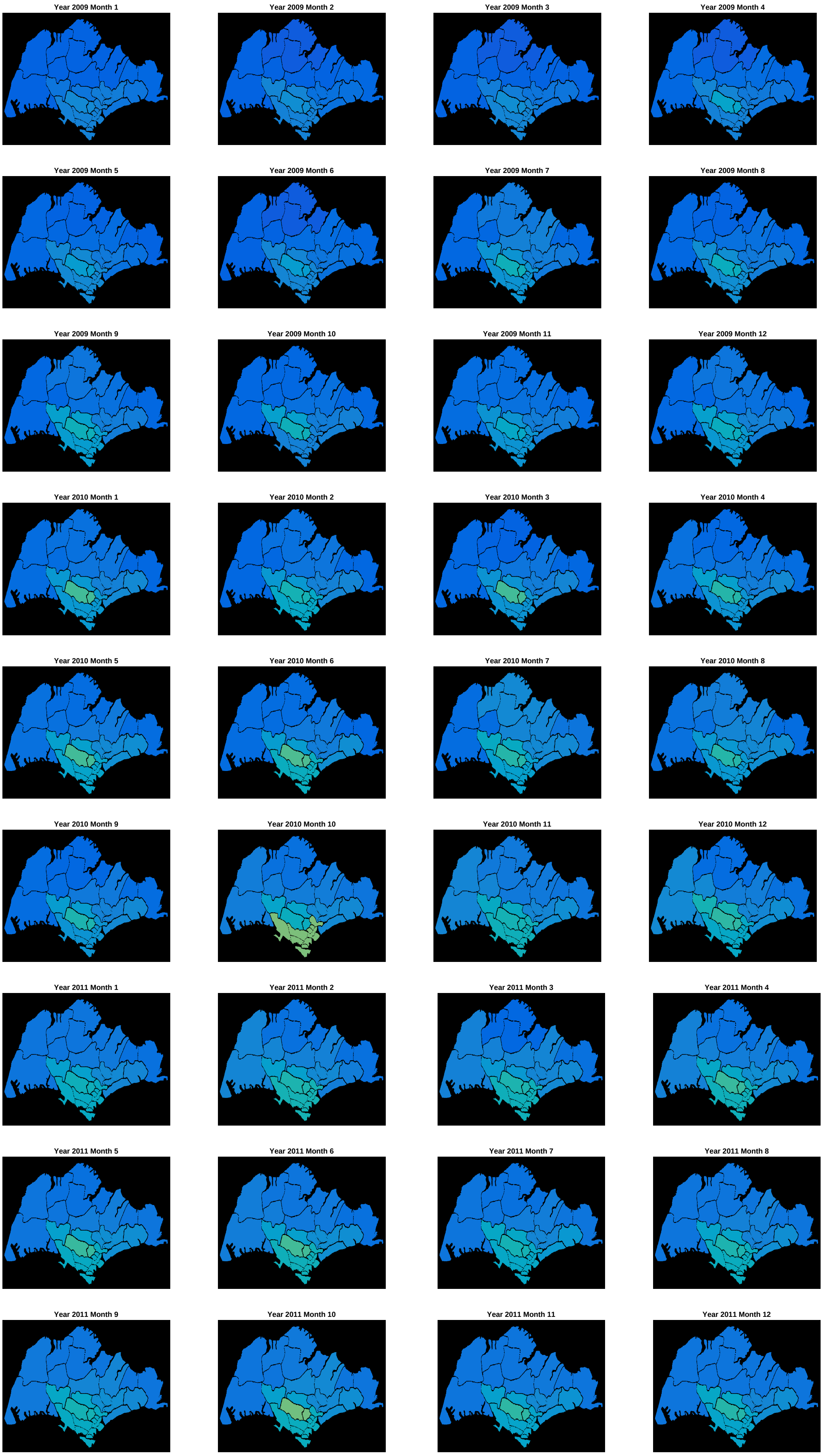

Figure 11: Landed properties Heat Map from 2009 to 2011

5 Dragon Kings and the U-test

Significant positive deviations from an empirically fitted distribution as detected by observation are indications of possible DKs. These DKs are a result from positive feedback mechanisms and are systematic deviations. Hence, these outliers cannot be confirmed just by looking at the p-value of the empirically fitted distribution. To test for the presence of DKs, we use the U-test proposed by Pisarenko and Sornette [10], but with modification for the use on the exponential distribution 1.

First, for our descending ordered empirical data set $X = \{x_1, x_2, \ldots, x_n\}$ we obtain the x_{min} and exponent $\hat{T}$ obtained from the MLE procedure discussed in **Section 1**. Using the estimated parameters x_{min} and $\hat{T}$ we obtained the estimated CDF, $F(x|\hat{T})$. By inserting the set X back into its CDF, we will obtain the uniform distribution [1], assuming the exponential distribution is a good estimate of the empirical data set X. Next, we note that for any empirical data set, we can define a empirical CDF,

$$P_n(x) = \frac{1}{n} \sum_{i=1}^{n} \mathbb{I}_{\{x_i < x\}}, \tag{8}$$

where $\mathbb{I}$ is the indicator function. If we randomly draw from this data set, the number of samples K, larger than x_i can be thought to be distributed by a binomial distribution $K \sim B(n, P_n(x))$, where n is the total number of data points. Analogously, the k-th ranked sample of a uniform distribution $U(0,1)$ would have the probability density function,

$$f_k(u) = \frac{n!}{(n-k)!(k-1)!} u^{k-1}(1-u)^{n-k}, \tag{9}$$

where u is the value of the observed k-th ranked sample.

The probability (or the p-value) of exceeding the value of u_k for each of the k-th sample is given as,

$$p(u_k) = \frac{n!}{(n-k)!(k-1)!} \int_{Z_k}^{1} w^{k-1}(1-w)^{n-k} dw \tag{10}$$

$$= 1 - betainc(Z_k, n-k+1, k), \tag{11}$$

where $Z_k = F(x_k|\hat{T})$ for the exponentially distributed X. Here, we measure the probability that the k-th ranked data point in the empirical distribution is deviated from uniform distribution. We follow Pisarenko and Sornette by classifying p-values with $p < 0.10$ as DKs [10].

References

[1] Housing Development Board. Buying a Flat [online]; 2005. Available from: `http://www.hdb.gov.sg/cs/infoweb/residential/buying-a-flat`. Accessed 01 Mar 2016.

[2] Singapore Land Authority. Foreign Ownership of Properties [online]; 2016. Available from: `http://www.sla.gov.sg/Services/RestrictiononForeignOwnershipofLandedProperty.aspx`. Accessed 30 May 2016.

[3] Clauset A, Shalizi CR, Newman ME. Power-law distributions in empirical data. SIAM Review. 2009;51(4):661–703.

[4] Forbes. Singapore 50 Richest 2015 Ranking [online]; 2015. Available from: `http://www.forbes.com/singapore-billionaires/list/`. Accessed 26 May 2016.

[5] Forbes. Taiwan 50 Richest 2015 Ranking [online]; 2015. Available from: `http://www.forbes.com/taiwan-billionaires/list/`. Accessed 26 May 2016.

[6] The Directorate General of Budget, Accounting and Statistics (DGBAS), Executive Yuan. Household Income Findings [in Chinese]; 2012. Available from: `http://win.dgbas.gov.tw/fies/index.asp`. Accessed 06 June 2016.

[7] Center for Survey Research, RCHSS, Academia Sinica. Survey of Family Income and Expenditure; 2012. Available from: `https://srda.sinica.edu.tw/gov/group_en/18`. Accessed 06 June 2016.

[8] Drăgulescu A, Yakovenko VM. Exponential and power-law probability distributions of wealth and income in the United Kingdom and the United States. Physica A: Statistical Mechanics and its Applications. 2001;299(1):213–221.

[9] Yakovenko V, Silva AC. Two-class structure of income distribution in the USA: exponential bulk and power-law tail. In: Chatterjee A, Yarlagadda S, Chakrabarti BK, editors. Econophysics of wealth distributions. Milano: Springer Milan; 2005. pp. 15–23.

[10] Pisarenko V, Sornette D. Robust statistical tests of Dragon-Kings beyond power law distributions. The European Physical Journal-Special Topics. 2012;205(1):95–115.

[1] This process is the reverse of Inverse transform sampling
