# Supplementary material for: Bubbles Are Departures from Equilibrium Housing Markets: Evidence from Singapore and Taiwan
Source: PLoS One. 2016 Nov 3;11(11):e0166004. doi: 10.1371/journal.pone.0166004 (PMC5094767; doi:10.1371/journal.pone.0166004)
Supplement: S2 File — This file contains additional information on the data sources used. The specific URLs and methods to obtain the data in parts or in whole is also contained within this document. (PDF) [file pone.0166004.s002.pdf]

Procedures for Obtaining Data Used in Bubbles are Departures from Equilibrium Housing Markets: Evidence from Singapore and Taiwan

1. Private Property Transaction Data from Real Estate Information System database

The private property transaction data in the Singapore housing market can be obtained from the Urban Redevelopment Authority's Real Estate Information System database in monthly files. These files can be queried with a valid subscription with REALIS.

Logging in to REALIS system:

Navigate to the REALIS Portal (https://spring.ura.gov.sg/lad/ore/login/index.cfm)

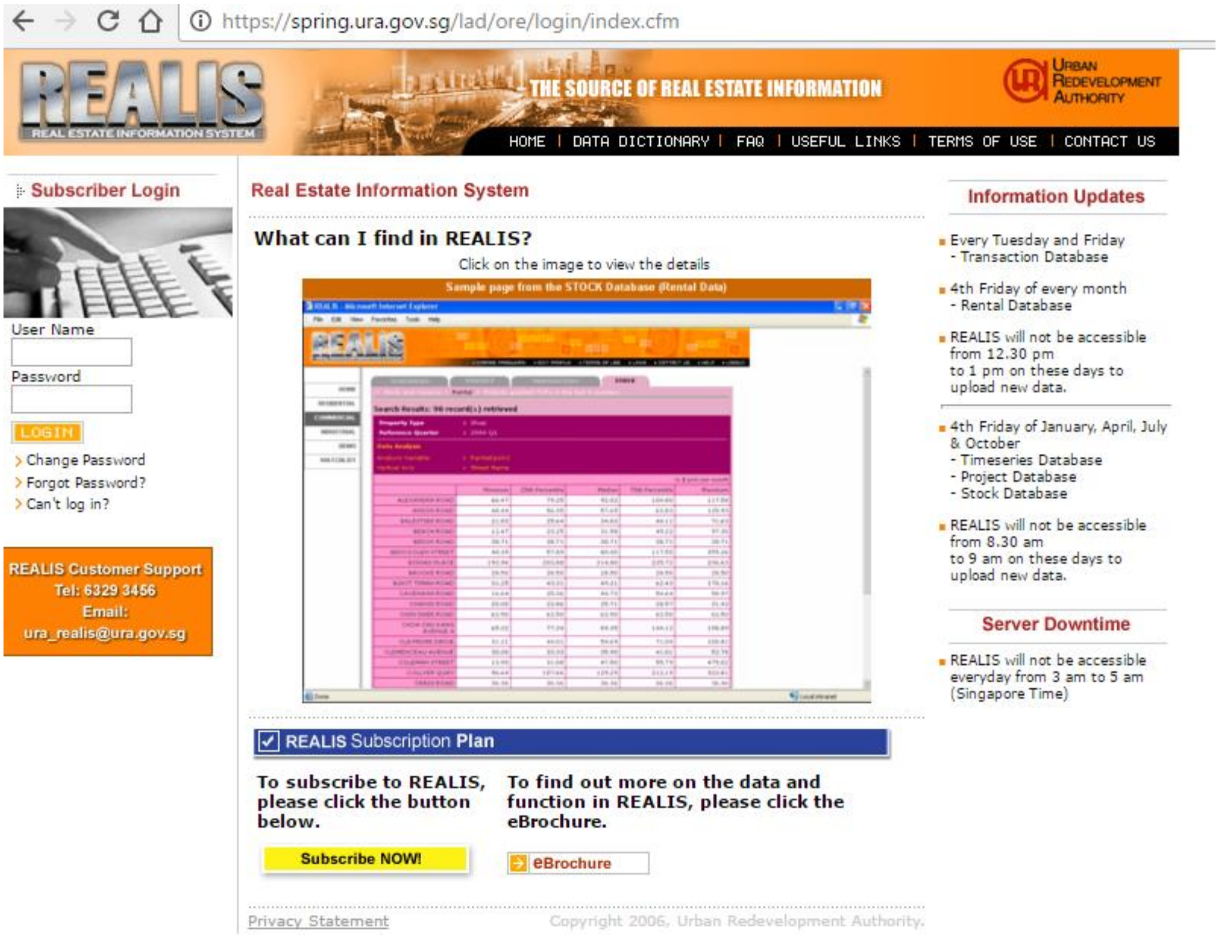

You will be prompted to accept the condition of use, click ‘here’ to proceed.

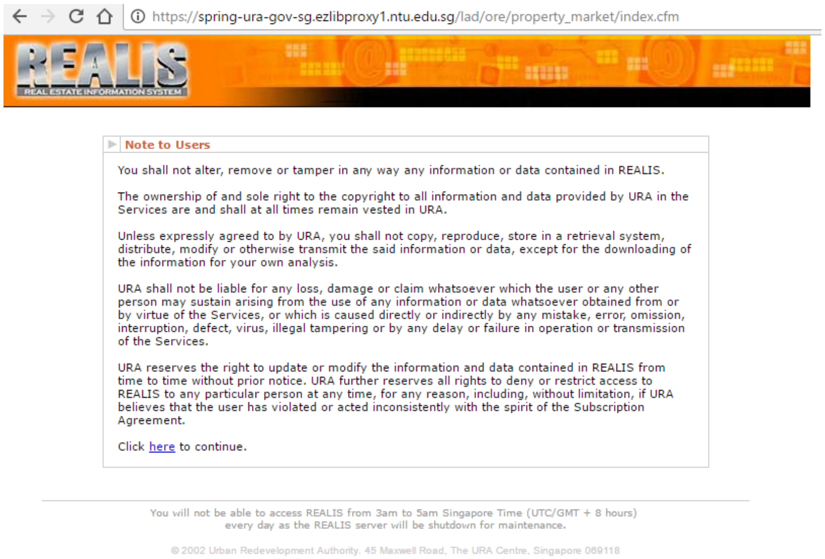
https://spring-ura-gov-sg.ezlibproxy1.ntu.edu.sg/lad/ore/property_market/index.cfm

REALIS
REAL ESTATE INFORMATION SYSTEM

Note to Users

You shall not alter, remove or tamper in any way any information or data contained in REALIS.

The ownership of and sole right to the copyright to all information and data provided by URA in the Services are and shall at all times remain vested in URA.

Unless expressly agreed to by URA, you shall not copy, reproduce, store in a retrieval system, distribute, modify or otherwise transmit the said information or data, except for the downloading of the information for your own analysis.

URA shall not be liable for any loss, damage or claim whatsoever which the user or any other person may sustain arising from the use of any information or data whatsoever obtained from or by virtue of the Services, or which is caused directly or indirectly by any mistake, error, omission, interruption, defect, virus, illegal tampering or by any delay or failure in operation or transmission of the Services.

URA reserves the right to update or modify the information and data contained in REALIS from time to time without prior notice. URA further reserves all rights to deny or restrict access to REALIS to any particular person at any time, for any reason, including, without limitation, if URA believes that the user has violated or acted inconsistently with the spirit of the Subscription Agreement.

Click here to continue.

You will not be able to access REALIS from 3am to 5am Singapore Time (UTC/GMT + 8 hours) every day as the REALIS server will be shutdown for maintenance.

© 2002 Urban Redevelopment Authority. 45 Maxwell Road, The URA Centre, Singapore 069118

On the left side tab, mouse over 'Residential', select 'Transaction'

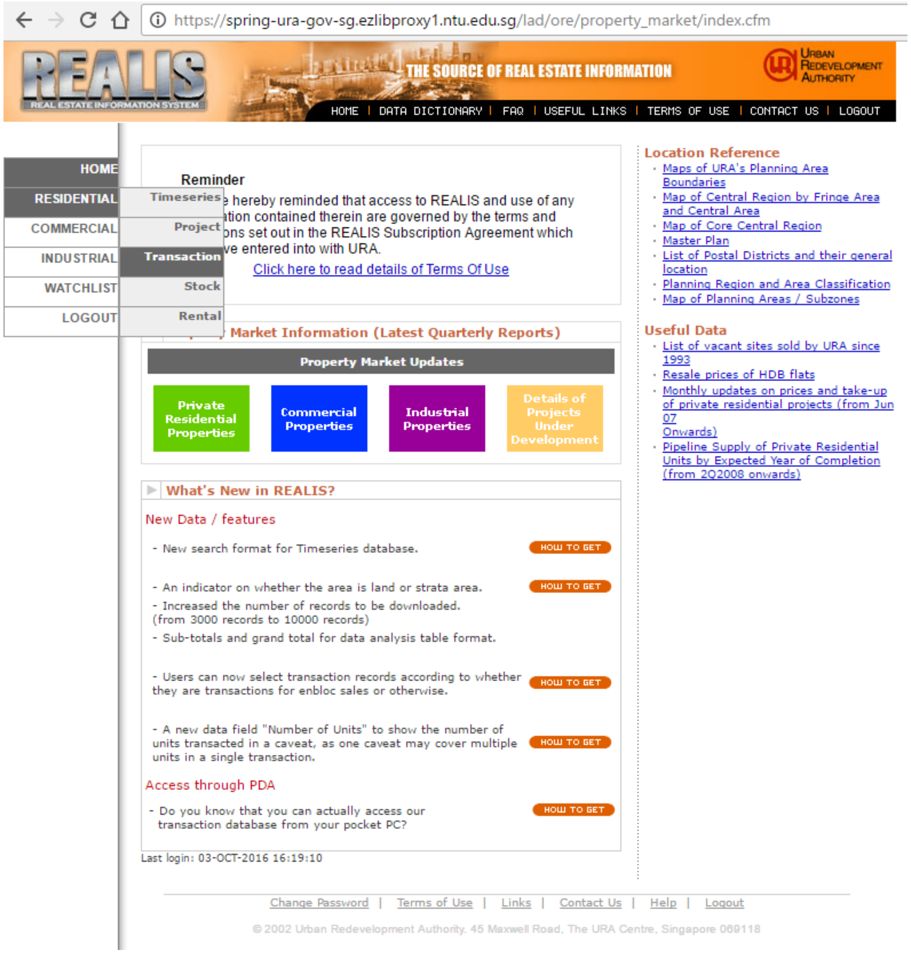

In the Property Type Field, select “All Property Type (including EC)” and select the desired “Sale Date”. Note that the maximum number of transaction that will be displayed per search is 10,000.

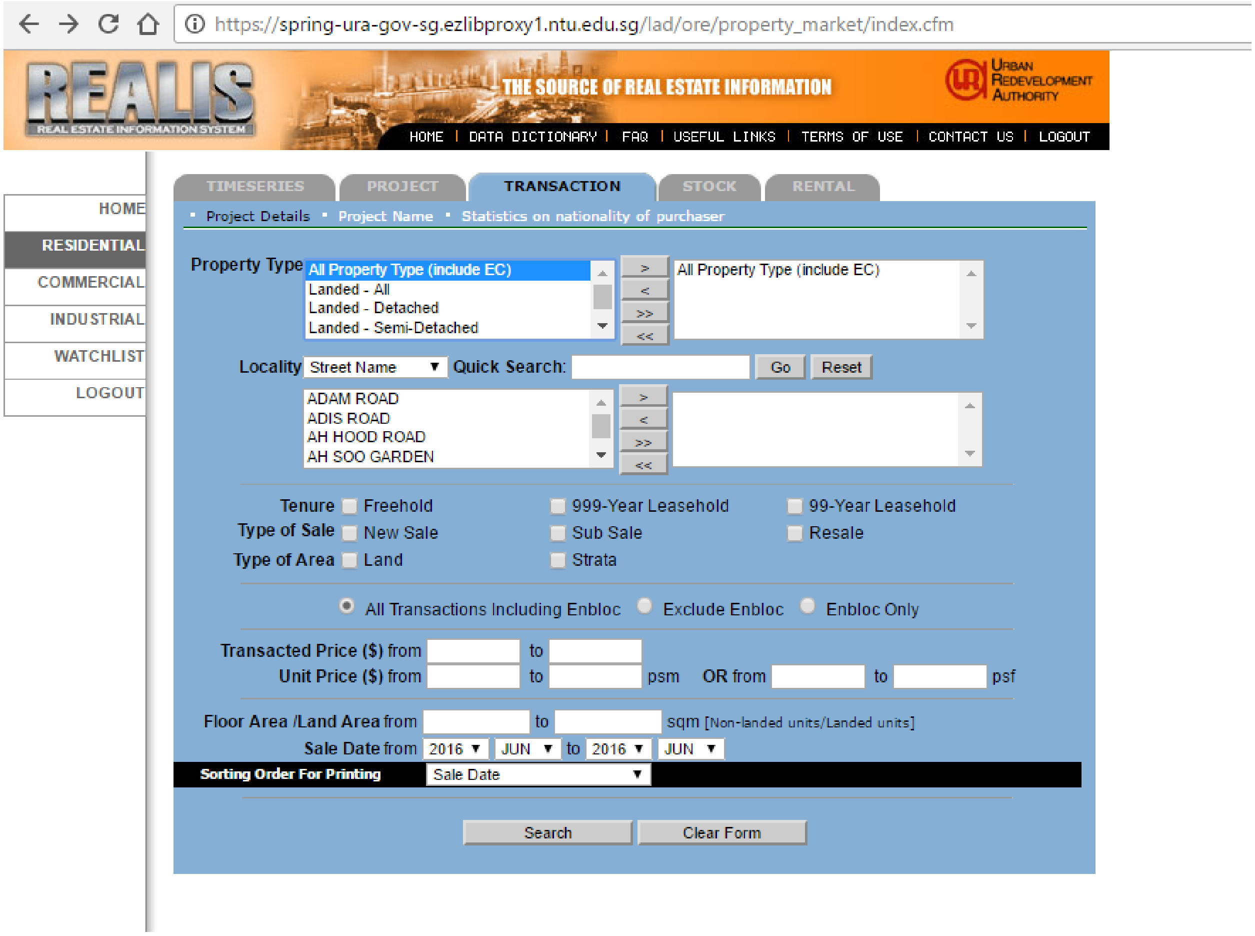

Scroll to the bottom of the Page when it loads and click on the “Download/Print Record” button to download the data.

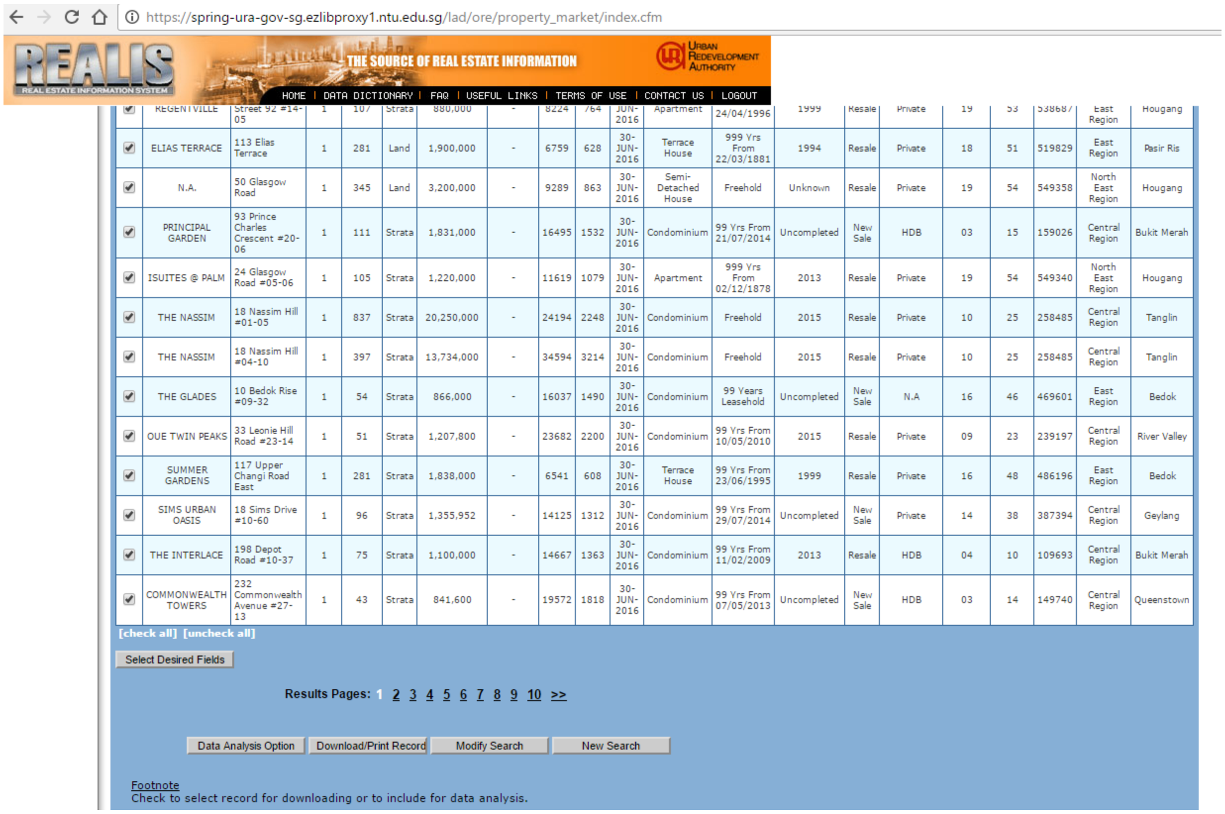

☑	REGENTVILLE	Street 92 #14-05	1	107	Strata	880,000	-	8224	764	JUN-2016	Apartment	24/04/1996	1999	Resale	Private	19	53	538687	East Region	Hougang
☑	ELIAS TERRACE	113 Elias Terrace	1	281	Land	1,900,000	-	6759	628	30-JUN-2016	Terrace House	999 Yrs From 22/03/1881	1994	Resale	Private	18	51	519829	East Region	Pasir Ris
☑	N.A.	50 Glasgow Road	1	345	Land	3,200,000	-	9289	863	30-JUN-2016	Semi-Detached House	Freehold	Unknown	Resale	Private	19	54	549358	North East Region	Hougang
☑	PRINCIPAL GARDEN	93 Prince Charles Crescent #20-06	1	111	Strata	1,831,000	-	16495	1532	30-JUN-2016	Condominium	99 Yrs From 21/07/2014	Uncompleted	New Sale	HDB	03	15	159026	Central Region	Bukit Merah
☑	ISUITES @ PALM	24 Glasgow Road #05-06	1	105	Strata	1,220,000	-	11619	1079	30-JUN-2016	Apartment	999 Yrs From 02/12/1878	2013	Resale	Private	19	54	549340	North East Region	Hougang
☑	THE NASSIM	18 Nassim Hill #01-05	1	837	Strata	20,250,000	-	24194	2248	30-JUN-2016	Condominium	Freehold	2015	Resale	Private	10	25	258485	Central Region	Tanglin
☑	THE NASSIM	18 Nassim Hill #04-10	1	397	Strata	13,734,000	-	34594	3214	30-JUN-2016	Condominium	Freehold	2015	Resale	Private	10	25	258485	Central Region	Tanglin
☑	THE GLADES	10 Bedok Rise #09-32	1	54	Strata	866,000	-	16037	1490	30-JUN-2016	Condominium	99 Years Leasehold	Uncompleted	New Sale	N.A	16	46	469601	East Region	Bedok
☑	OUE TWIN PEAKS	33 Leonie Hill Road #23-14	1	51	Strata	1,207,800	-	23682	2200	30-JUN-2016	Condominium	99 Yrs From 10/05/2010	2015	Resale	Private	09	23	239197	Central Region	River Valley
☑	SUMMER GARDENS	117 Upper Changi Road East	1	281	Strata	1,838,000	-	6541	608	30-JUN-2016	Terrace House	99 Yrs From 23/06/1995	1999	Resale	Private	16	48	486196	East Region	Bedok
☑	SIMS URBAN OASIS	18 Sims Drive #10-60	1	96	Strata	1,355,952	-	14125	1312	30-JUN-2016	Condominium	99 Yrs From 29/07/2014	Uncompleted	New Sale	Private	14	38	387394	Central Region	Geylang
☑	THE INTERLACE	198 Depot Road #10-37	1	75	Strata	1,100,000	-	14667	1363	30-JUN-2016	Condominium	99 Yrs From 11/02/2009	2013	Resale	HDB	04	10	109693	Central Region	Bukit Merah
☑	COMMONWEALTH TOWERS	232 Commonwealth Avenue #27-13	1	43	Strata	841,600	-	19572	1818	30-JUN-2016	Condominium	99 Yrs From 07/05/2013	Uncompleted	New Sale	HDB	03	14	149740	Central Region	Queenstown

[check all] [uncheck all]

Select Desired Fields

Results Pages: 1 2 3 4 5 6 7 8 9 10 >>

Data Analysis Option | Download/Print Record | Modify Search | New Search

Footnote
Check to select record for downloading or to include for data analysis.

2. Housing Development Board Flats Transaction

This data set can be downloaded from the Singapore public data site:

https://data.gov.sg/dataset/resale-flat-prices

The Resale Flat Prices (Based on Approval Date), 2000 – Feb 2012, with the specific link to the file included below:

https://data.gov.sg/dataset/7a339d20-3c57-4b11-a695-9348adfd7614/resource/8c00bf08-9124-479e-aeca-7cc411d884c4/download

3. Household Findings of Taiwan

This data set has been pre-processed and can be downloaded free from the website of the Directorate-General of Budget, Accounting and Statistics, Executive Yuan, R.O.C and can be accessed at:

http://win.dgbas.gov.tw/fies/index.asp

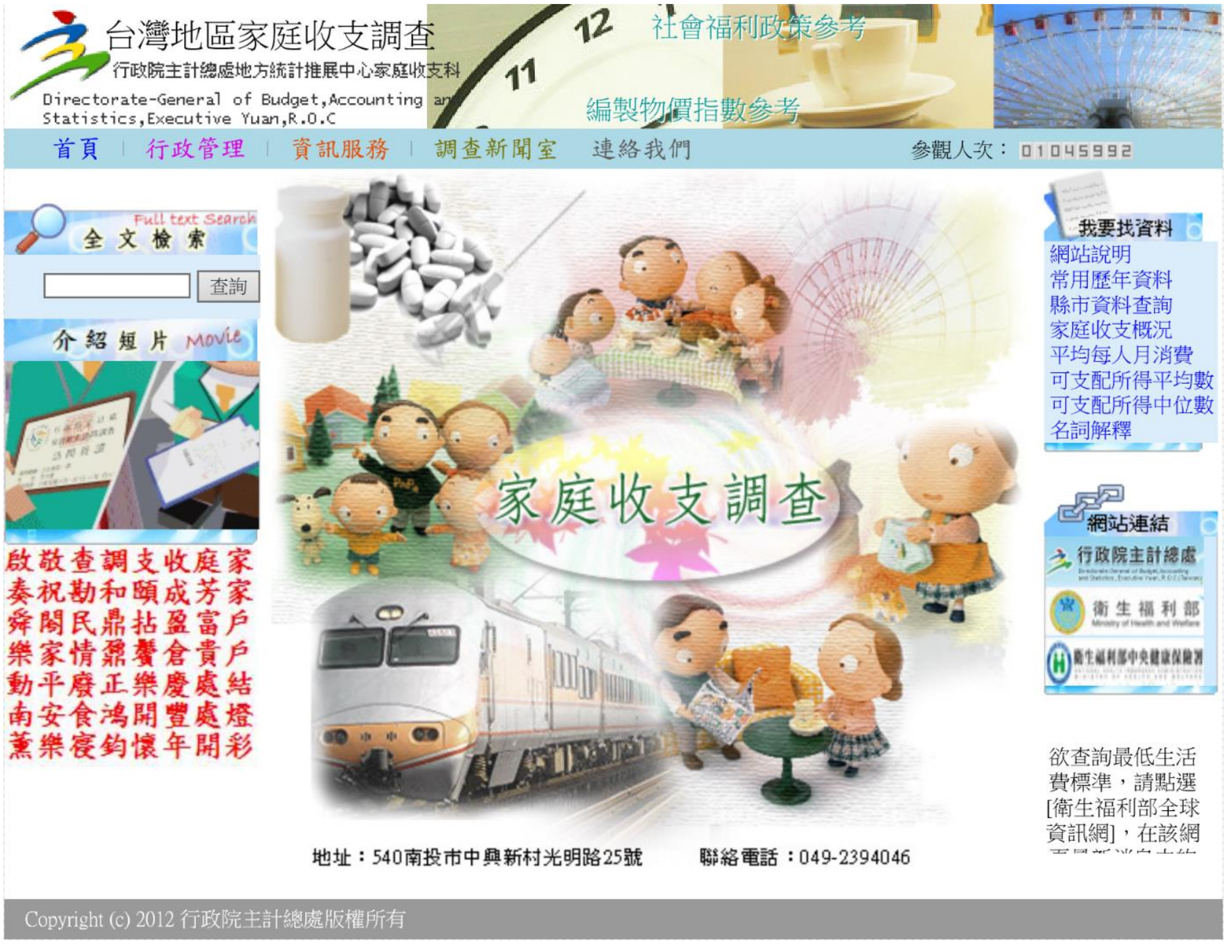

One can click the items on the right column, e.g., “常用歷年資料” gives you the historical data for the average family income and expenditure per household by areas from 1981 till 2015.

For the raw data, one needs to fill out a form and pay for the data set. The website can be accessed at:

http://win.dgbas.gov.tw/fies/order.asp

To download the form, click on the phrase “統計資訊申請單”. Fill out the form and fax to 886- 049-2394040.

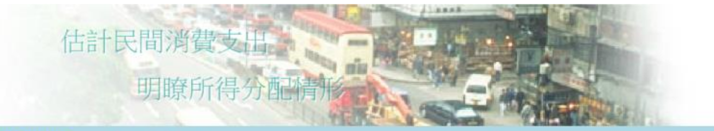

資訊服務

資料申購

資料格式範例
資料格式說明
歷年家庭收支調查統計分類編號表
歷年家庭收支調查各科目總額表、平均每戶表、平均每人表
資料讀取SAS程式範例

若您需購買本總處之家庭收支調查電腦媒體原始資料，可逕填寫本總處提供統計資訊申請單，傳真至(049)2394040即可。
如有資料相關問題，請洽本總處地方統計推展中心家庭收支科「黃麗妃」小姐 Tel:(049) 2394046。

Copyright (c) 2012 行政院主計總處版權所有

<u>4. Real Estate Transaction file of Taiwan</u>

This data set can be downloaded from the Department of Land Administration following the steps below:

First access the Department of Land Administration open data webpage below:

http://plvr.land.moi.gov.tw/DownloadOpenData

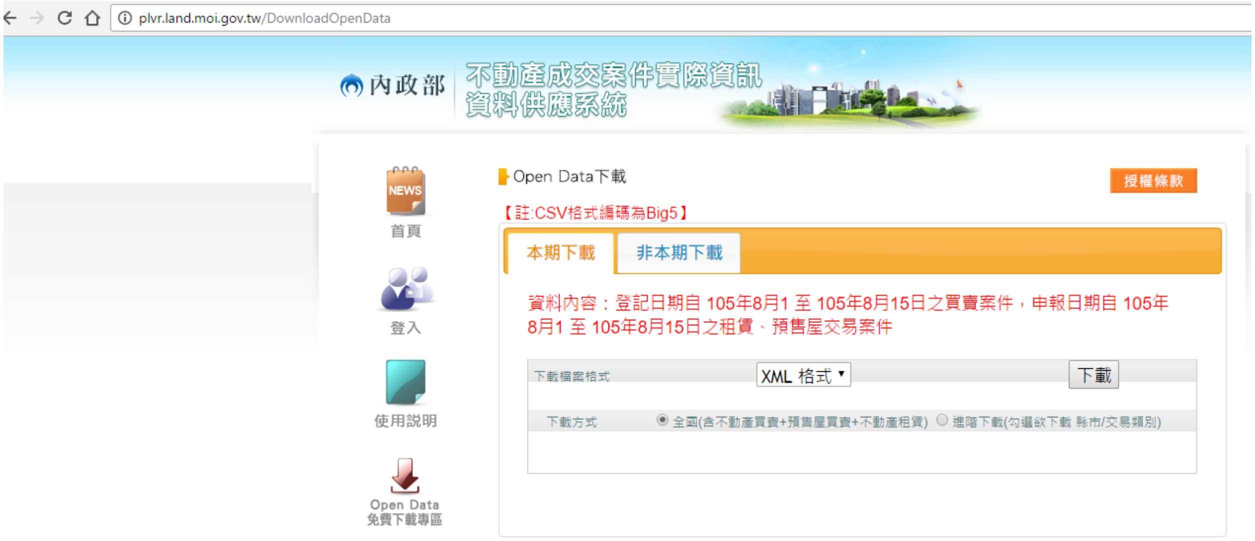

The transactions records are published regularly every quarter. To download historical data:

1) select the option “非本期下载”
2) On the drop down list, select the Year and quarter that you would like to download. Note that the year quoted here is the Minguo year. To convert it to the Gregorian calendar year, add 1911 to the Minguo year. In the example below we are downloading, “105 年第 3 季” which in the Gregorian calendar is Year 2016 (105 + 1911), 3rd quarter
3) Click on the “下载” button to download the data*

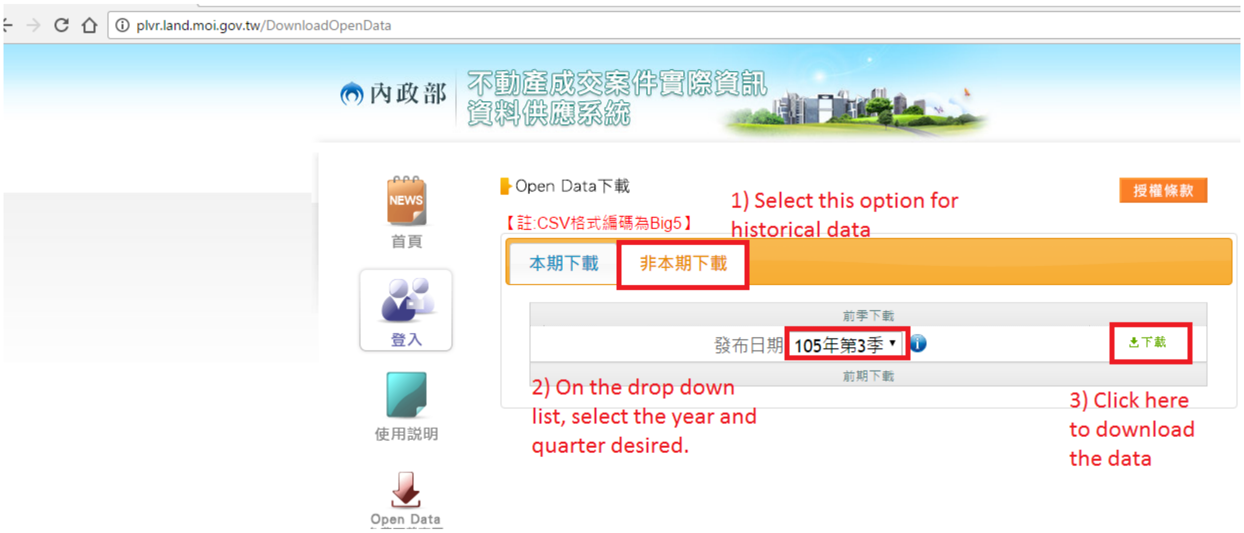

*Note that the downloaded file in csv format uses the Big5 encoding and comma delimited.
